# Supplementary material for: High-Q lasing via all-dielectric Bloch-surface-wave platform
Source: Nat Commun. 2023 Oct 13;14:6458. doi: 10.1038/s41467-023-41471-8 (PMC10576087; doi:10.1038/s41467-023-41471-8)
Supplement: Supplementary file 1 — Supplementary Information [file 41467_2023_41471_MOESM1_ESM.pdf]

Supplementary Information

High-Q lasing via all-dielectric Bloch-surface-wave platform

*Yang-Chun Lee[1], Ya-Lun Ho[1], Bo-Wei Lin[1], Mu-Hsin Chen[1], Di Xing[1], Hirofumi Daiguji[1], Jean-Jacques Delaunay[1]**

[1]Department of Mechanical Engineering, School of Engineering

The University of Tokyo

7-3-1 Hongo, Bunkyo-ku, Tokyo 113-8656, Japan

E-mail: jean@mech.t.u-tokyo.ac.jp

Supplementary Table 1. Lasers based on guiding surface wave modes.

Gain medium (method)	Top-down fabrication	Waveguide mode (guiding structure)	FWHM	Year	Ref.
CdS nanowire (CVD)	No	Hybrid plasmonic (CdS-MgF_2-Ag film)	0.5 nm	2009	[1]
InGaN@GaN nanowire (plasma-assisted MBE)	No	Hybrid plasmonic (GaN-SiO_2-Ag film)	0.5 nm	2012	[2]
GaN nanowire (MOCVD)	No	Hybrid plasmonic (GaN-SiO_2-Al film)	0.8 nm	2014	[3]
ZnO nanowire (VLS technique)	No	Hybrid plasmonic (ZnO-LiF-Ag film)	~1 nm	2014	[4]
$MAPbI_3$ nanowire (solution growth)	No	Hybrid plasmonic ($MAPbI_3$-MgF_2-Ag film)	5.2 nm	2016	[5]
ZnO nanowire (hydrothermal method)	No	Plasmonic (ZnO-Al film)	0.2 nm	2016	[6]
CdSe nanosquare (CVD)	No	Hybrid plasmonic (CdSe-MgF_2-Au film)	0.7 nm	2017	[7]
DCM doped PMMA (top-down fabrication)	Yes	Hybrid plasmonic (DCM-Al_2O_3-Ag film)	1.8 nm	2017	[8]
GaAs nanowire (MBE)	No	Plasmonic (GaAs-Au VG channel)	~1 nm	2017	[9]
$CsPbBr_3$ nanowire (CVD)	No	Hybrid plasmonic ($CsPbBr_3$-SiO_2-Ag film)	0.7 nm	2018	[10]
IR-140 doped PMMA (top-down fabrication)	Yes	LRSPP[a)] (IR-140-Ag DFB[b)] grating)	0.2 nm	2018	[11]
$MAPbI_3$ microplate (solution growth)	No	Hybrid plasmonic ($MAPbI_3$-SiO_2-Au film)	~2 nm	2018	[12]
ZnO waveguide (top-down fabrication)	Yes	Plasmonic (ZnO-Al layer)	1.4 nm	2018	[13]
ZnO nanowire (hydrothermal method)	No	Hybrid plasmonic (ZnO-graphene-Al_2O_3-Ag film)	0.5 nm	2019	[14]
$CsPbBr_3$ crystals (solution growth)	No	Hybrid plasmonic ($CsPbBr_3$-pNE-Au film)	0.2 nm	2021	[15]
$CsPbBr_3$ quantum dot (solution growth)	No	Hybrid plasmonic ($CsPbBr_3$-PVP-Ag nanowire)	0.4 nm	2021	[16]
$MAPbBr_3$ nanowire (solution growth)	No	Hybrid plasmonic ($MAPbBr_3$-MgF_2-Ag film)	~1 nm	2021	[17]
R6G doped SU-8 (top-down fabrication)	Yes	BSW (R6G-dielectric multilayer)	0.02 nm	2022	This work

a) LRSPP, long-range surface plasmon polariton

b) DFB, distributed feedback

Supplementary Table 2. On-chip WGM lasers based on dye-doped polymers.

Gain medium (method)	Waveguide mode	Guiding structure thickness	Diameter	Threshold	Pulse duration of pump laser	Year	Ref.
BEH-PPV	Photonic mode	NA	100 μm	10 μJ/mm^2	10 ns	1998	[18]
R6G doped SU-8	Photonic mode	48 μm	(trapezoid) round-trip length 292 μm	25 μJ/mm^2	5 ns	2005	[19]
R6G doped PMMA	Photonic mode	1 μm	40 μm	2.5 μJ/mm^2	15 ns	2010	[20]
PM597 doped resist	Photonic mode	1 μm	47 μm	12 μJ/mm^2*	5 ns	2011	[21]
PM597 doped PMMA	Photonic mode	~1 μm	50 μm	0.3 μJ/mm^2	20 ns	2015	[22]
R6G doped SU-8	Photonic mode	12 μm	300 μm	0.2 μJ/mm^2	10 ns	2015	[23]
R6G-doped TZ-001	Photonic mode	30 μm	220 μm	9.3 μJ/mm^2	5 ns	2017	[24]
R6G doped SU-8	Photonic mode	30 μm	220 μm	2.5 μJ/mm^2	5 ns	2018	[25]
R6G doped SU-8	Photonic mode	0.24 μm (at the cutoff of the photonic mode)	100 μm	100 μJ/mm^2	0.5 ns	2023	This work
R6G doped SU-8	BSW mode	0.14 μm	100 μm	6.7 μJ/mm^2	0.5 ns	2023	This work

*The pumped spot size is assumed to be 50 μm

Optical properties of the Rhodamine 6G laser dye

Supplementary Figure 1 displays the absorbance spectrum of the Rhodamine 6G (R6G) dye in ethanol/SU-8 solution having a dye concentration of 0.2 mM and the photoluminescence (PL) spectrum of the cured R6G-doped (1 mM) SU-8 polymer layer having a thickness of 520 nm on quartz. The peak of absorbance of the R6G dye is located at about 532 nm, and a PL peak is observed which is located at approximately 560 nm. The lasing band observed in the R6G-doped SU-8 ring lasers is also highlighted, with a roughly centered wavelength of 580 nm.

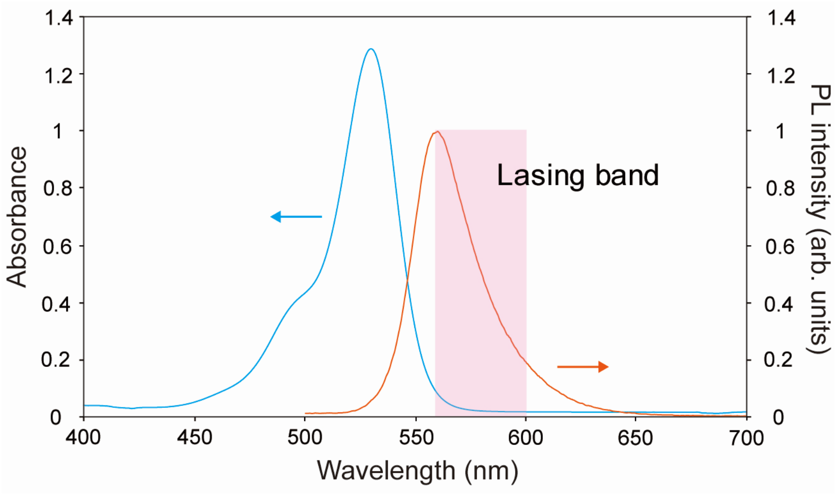

Supplementary Figure 1. Absorbance spectrum of the R6G dye (0.2 mM) in ethanol/SU-8 solution and the PL spectrum of the R6G-doped SU-8 polymer layer on quartz.

Design of the dielectric multilayer sustaining the Bloch surface waves (BSWs).

The dielectric multilayer sustains BSW modes at wavelengths of the lasing band from R6G-doped SU-8 ring lasers. In this work, a BSW platform consisting of five pairs of alternating TiO_2 (high refractive index, n = 2.31) and SiO_2 (low refractive index, n = 1.46) layers was designed for the wavelength of 580 nm. The layer respective thicknesses d are calculated based on the equation[26]:

$$d = \frac{\lambda}{4n\cos\theta} \quad \text{(S1)},$$

where λ is the designed wavelength, n the refractive index, and θ the refraction angle of light within the TiO_2 and SiO_2 layers, respectively. According to this equation, the thicknesses of TiO_2 and SiO_2 layers are 71 and 154 nm, respectively, which are designed at refraction angle θ of 50° within the SiO_2 layer (thus, the refraction angle of light within the TiO_2 layer is 29°). Supplementary Figure 2 displays the BSW dispersion diagram of the TiO_2/SiO_2 multilayer having respective thicknesses of 71 and 154 nm with a SU-8 layer on the top of the multilayer. The forbidden band of the TiO_2/SiO_2 multilayer is displayed, and the wavelength range corresponding to the lasing band of the R6G-doped SU-8 ring lasers is also indicated. In Supplementary Figure 2, the BSW modes of the multilayer with different SU-8 top layers are located in the forbidden band, and, furthermore, all of the BSW modes having wavelengths located in the lasing band can be obtained when the thickness of the planar SU-8 layer is less than 200 nm. Accordingly, it suggests that the designed dielectric multilayer indeed sustains BSW modes for the range of wavelengths of interest.

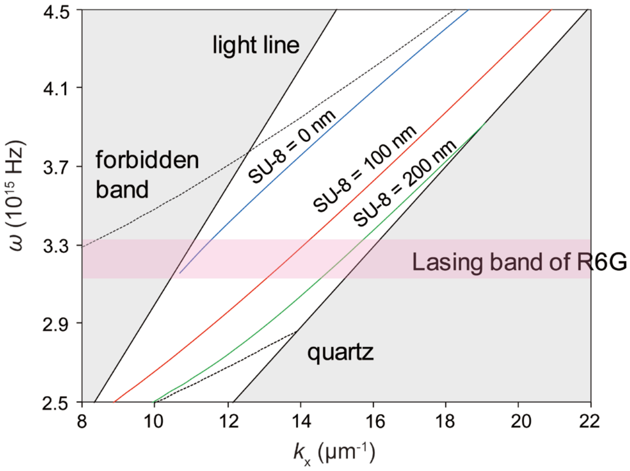

Supplementary Figure 2. The BSW dispersion diagram of the TiO_2/SiO_2 multilayer with respective thicknesses of 71 and 154 nm without (0 nm, blue) and with (100 and 200 nm, red and green, respectively) a top SU-8 polymer layer.

The effect of the ridge width on the photonic, the hybrid plasmonic, and the BSW mode

The effect of the ridge width on the optical characteristics of the photonic, the hybrid plasmonic, and the BSW mode are also investigated. Supplementary Figures 3a–c display the calculated real part (Supplementary Figure 3a) and imaginary part (Supplementary Figure 3b) of the mode effective indices ($n_{\text{effective}} + ik_{\text{effective}}$) and the confinement factor (Supplementary Figure 3c) of the guided photonic, the hybrid plasmonic, and the BSW mode as a function of the ridge width. Here, the thicknesses of the ridge waveguides are set to be 240/140/140 nm, which support photonic/hybrid plasmonic/BSW mode, respectively. In Supplementary Figures 3a–c, the $n_{\text{effective}}$, $k_{\text{effective}}$, and the confinement factor of these three guided modes all exhibit a weak dependence when the ridge width is larger than 1 μm. When the ridge width is further decreased, they demonstrate different behaviors. For the photonic mode, a mode cutoff, where the confinement factor drops rapidly, can be observed when the ridge is smaller than 700 nm. For the hybrid plasmonic mode, on the other hand, good optical confinement over the ridge width is obtained. Furthermore, for the BSW mode, the confinement factor starts to decrease rapidly when the ridge width is smaller than 800 nm, and the $k_{\text{effective}}$ (corresponding to propagation loss) increases from 10^{-4} to10^{-3}. In Supplementary Figure 3b, an important feature worth noticing is revealed: the BSW mode experiences smaller propagation loss ($k_{\text{effective}}$) than the hybrid plasmonic mode for a range of the ridge width from 0.5 to 2.5 μm, indicating an important advantage of the BSW-based devices for long-range light propagation.

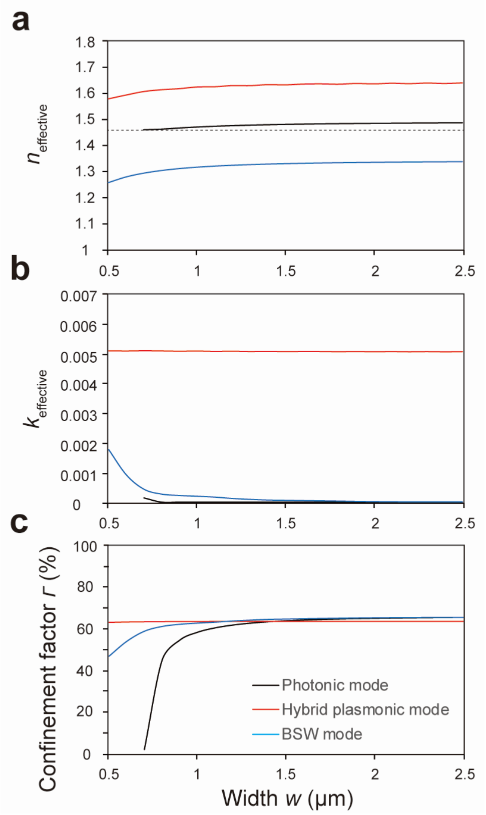

Supplementary Figure 3. Optical characteristics of photonic (d = 240 nm), hybrid plasmonic (d = 140 nm), and BSW (d = 140 nm) modes as a function of the SU-8 polymer ridge width w. (a, b) The (a) real part and (b) imaginary part of the mode effective indices (expressed by $n_{effective} + ik_{effective}$). (c) The confinement factor. The BSW platform comprises five TiO_2/SiO_2 pairs having respective thicknesses of 71 nm/154 nm.

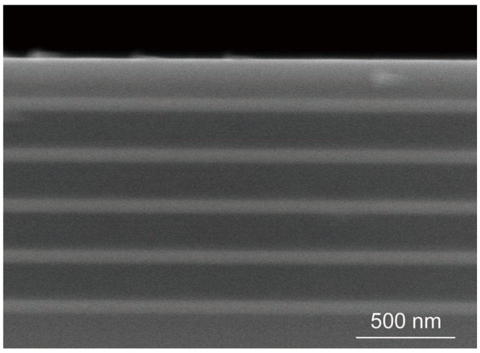

Supplementary Figure 4. Cross-sectional SEM image of the dielectric multilayer fabricated on a silicon substrate. The dielectric multilayer serving as the BSW platform comprised five pairs of alternating TiO_2 and SiO_2 layers having respective actual thicknesses of 82 and 170 nm.

The effect of the ridge oblique sidewall on the optical characteristics of the BSW mode

Since the R6G-doped SU-8 polymer ridge shows non-vertical sidewalls in SEM and AFM images, the effect of the oblique sidewall using mode analysis was investigated. Supplementary Figures 5a and 5b show the AFM images of the R6G-doped SU-8 ridges of the BSW ring cavity (d = 136 nm and w = 1.5 μm) and photonic ring cavity (d = 240 nm and w = 1.5 μm), respectively, and their height profiles are displayed in Supplementary Figure 5c. From the AFM results, the sidewall angle of the ridge of the BSW ring cavity is about 16.8°, and that of the photonic ring cavity is about 42°. Accordingly, the simulation models are adjusted to investigate the sidewall effect on the optical characteristics of the guided modes.

Supplementary Figures 6a–c display the calculated $n_{\text{effective}}$ (Supplementary Figure 6a), $k_{\text{effective}}$ (Supplementary Figure 6b), and confinement factor (Supplementary Figure 6c) of the BSW mode as a function of width with vertical (red line) and non-vertical (blue line) sidewalls. The SU-8 ridge thickness is 140 nm. In Supplementary Figures 6a–c, the oblique sidewall has almost no effect on the $n_{\text{effective}}$ of the BSW mode. In contrast, the $k_{\text{effective}}$ and the confinement factor of the BSW modes are slightly influenced by the small ridge width. Such an increase in the $k_{\text{effective}}$ and decrease in the confinement factor suggest that the propagating light tends to leak into the substrate due to less optical confinement. However, for a SU-8 polymer ridge having a width larger than 1.5 μm, the propagation loss of the BSW mode ($k_{\text{effective}} < 10^{-4}$) is still significantly lower than those of the hybrid plasmonic mode ($k_{\text{effective}} \sim 5\times10^{-3}$).

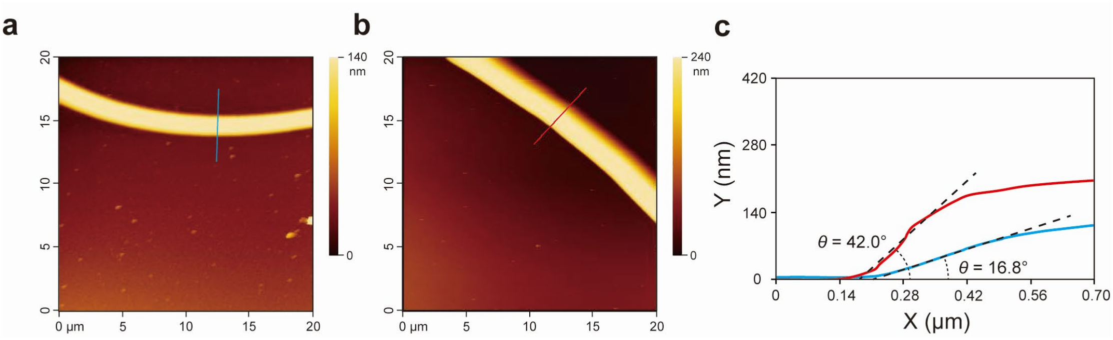

Supplementary Figure 5. AFM images of the R6G-doped SU-8 ridges of (a) BSW ring cavity with d = 136 nm and w = 1.5 μm and (b) photonic ring cavity with d = 240 nm and w = 1.5 μm. (c) The magnified height profile along the solid lines.

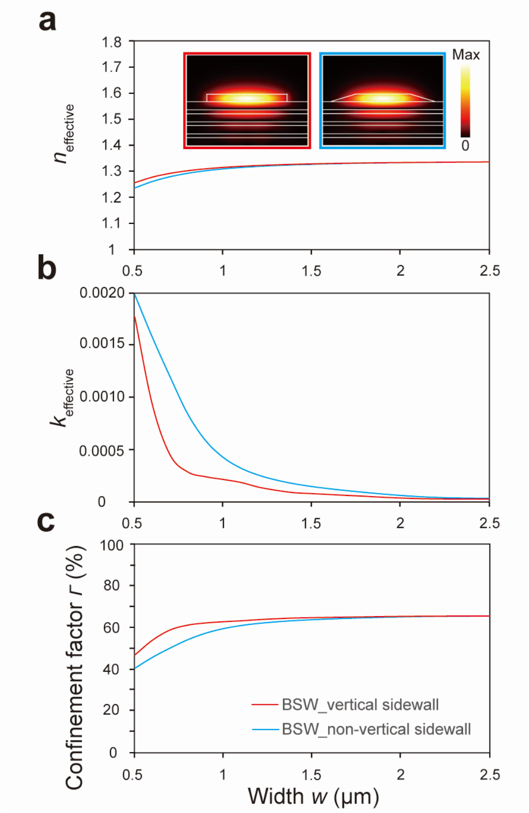

Supplementary Figure 6. Optical characteristics of BSW modes as a function of the SU-8 polymer ridge width w with vertical (red line) and non-vertical (blue line) sidewalls (d = 136 nm). (a, b) The (a) real part and (b) imaginary part of the mode effective indices (expressed by $n_{\text{effective}} + ik_{\text{effective}}$). Inset in figure a: the calculated electric field distributions of the BSW modes for d = 136 nm and w = 1.5 μm. (c) The confinement factor. The BSW platform comprises five TiO_2/SiO_2 pairs having respective thicknesses of 71 nm/154 nm.

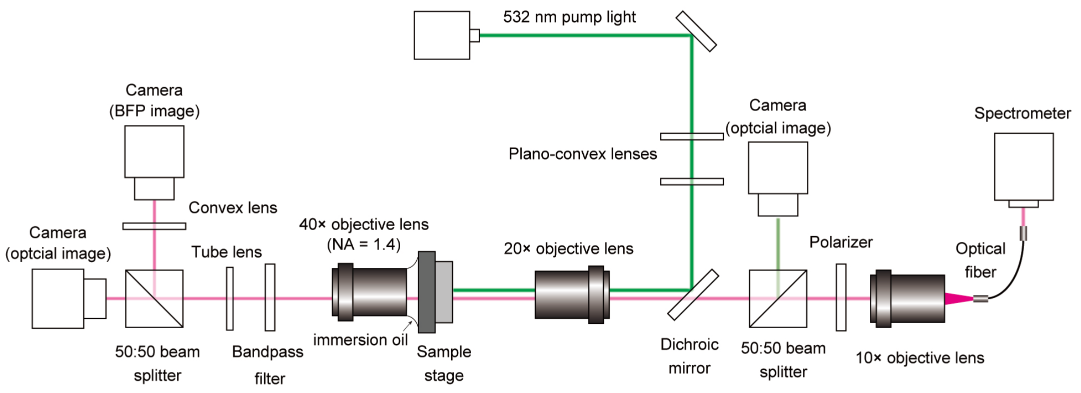

Supplementary Figure 7. Schematic representation of the experimental setup for laser performance measurement. A nanosecond (ns) pulsed laser having a wavelength of 532 nm was used as the pump light. The pump light was focused on the microring cavities using a 20× objective lens with a numerical aperture (NA) of 0.45. For laser performance measurements, the ring cavities were optically pumped and their emissions collected by the same 20× objective lens. The emitted light was then split by a 50:50 beam splitter and either collected into a charge-coupled device (CCD) camera using a tube lens for optical image observation or collected using a 10× objective lens and guided into the spectrometer using an optical fiber for spectral analysis. For leakage radiation microscopic measurements, the leakage radiation from the sample back (substrate side) was also collected with an oil immersion objective lens having a numerical aperture of 1.4. A band-pass filter with a central wavelength of 580 nm and a full width at half maximum (FWHM) of 10 nm was used to only filter out the leakage radiation light from the stimulated process. Then, the leakage radiation was transmitted through a tube lens and split by a 50:50 beam splitter. The leakage radiation was then either captured by a CCD camera for optical image observation or collected using a convex lens to produce a back focal plane (BFP) image on a CCD camera (corresponding to the Fourier transform of the direct plane image). By analyzing the BFP image of the BSW lasers, the wave-vector information (e.g., the effective refractive index) of the lasing mode was then obtained.

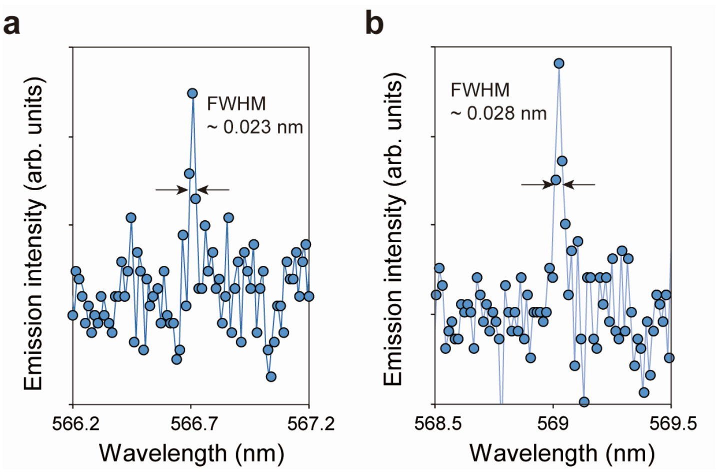

Supplementary Figure 8. The magnified emission spectra of BSW lasers having (a) d = 136 nm, w = 1.5 µm, and D = 50 µm and (b) d = 136 nm, w = 1.5 µm, and D = 30 µm. Note that the FWHMs are slightly larger than the spectrometer spectral resolution.

Discussion of the group index of the BSW lasers

The group index n_g of the BSW lasers is theoretically calculated from the BSW dispersion diagram (Supplementary Figure 9a) and is compared to the experimentally determined value. Here, the BSW dispersion was obtained using the mode analysis. The group index is defined as the ratio of the velocity of light in vacuum (c) to the group velocity (v_g) for the BSW mode. The group velocity v_g is expressed by:

$$v_g = \frac{d\omega}{dk} \quad \text{(S2)},$$

which can be directly obtained from the slope of the BSW dispersion. Accordingly, the group index n_g of the BSW laser is calculated to be 1.83 from the definition $n_g = c/v_g$, and this value agrees well with the experimentally determined group index of 1.82.

For comparison purposes, the group index n_g of the photonic lasers is also theoretically and experimentally investigated. First, the dispersion diagram of the SU-8 polymer ridge on a quartz substrate is calculated using the mode analysis, as displayed in Supplementary Figure 9a. According to the definition, the group index n_g of the photonic lasers is estimated to be 1.72. On the other hand, to determine the group index n_g experimentally, photonic lasers (d = 520 nm) with different diameters ranging from 30 to 100 μm were optically pumped, and their lasing mode spacing $\Delta\lambda$ versus the inverse of the cavity dimension $1/(\pi D)$ is plotted in Supplementary Figure 9c. From the linear dependence between $\Delta\lambda$ and $1/(\pi D)$, the group index ng of the photonic lasers is estimated to be 1.70 using the theoretical prediction $\Delta\lambda = \lambda^2/(\pi D n_g)$. Note that this value agrees well with the theoretical value, and is different from that of the BSW lasers (Supplementary Figure 9b). According to the investigation of the group index, lasing from the BSW lasers shows distinguishable characteristics from the photonic lasers, confirming that the lasing action of the BSW lasers is based on the stimulated emission of a BSW mode.

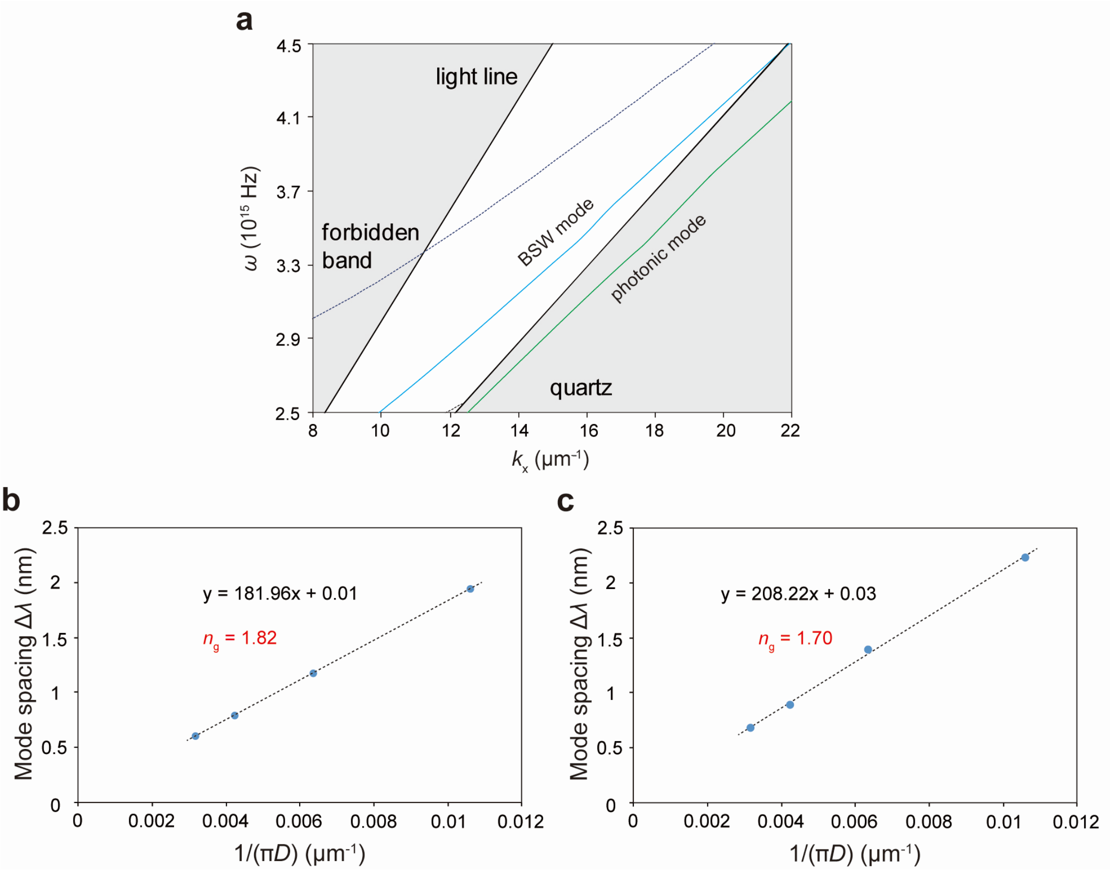

Supplementary Figure 9. The investigation of group index. (a) The BSW dispersion diagram of the TiO_2/SiO_2 multilayer having respective thicknesses of 82 and 170 nm with a 136-nm-thick top SU-8 polymer ridge (with non-vertical sidewall, blue). The dispersion diagram of the SU-8 polymer ridge on a quartz substrate (having a thickness of 520 nm and non-vertical sidewall in green) is also displayed. (b, c) Mode spacing analysis of the (b) BSW lasers and the (c) photonic lasers (d = 520 nm) as a function of their cavity dimensions. The mode spacing is inversely proportional to the diameter of the ring cavity.

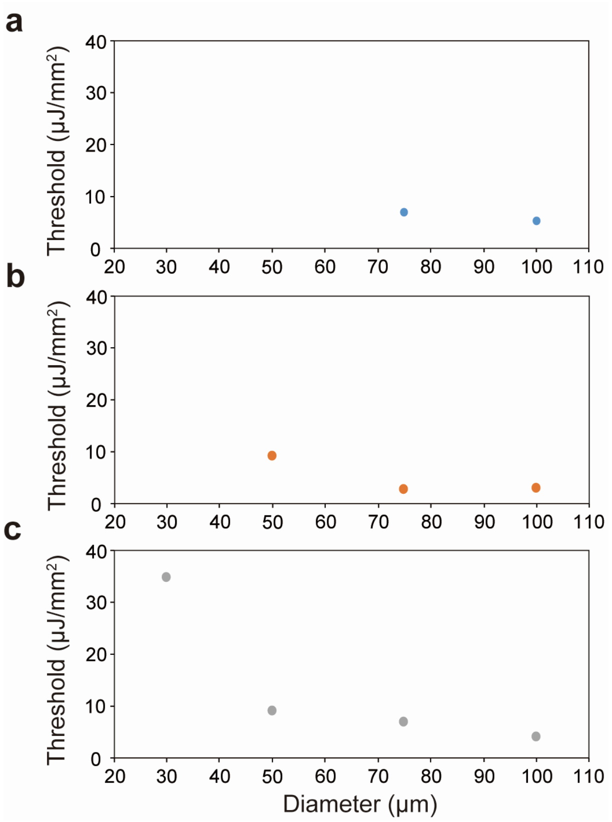

Supplementary Figure 10. Measured threshold values of the ring cavities having different diameters *D* on quartz substrate (photonic lasers). (a) Thickness *d* = 290 nm, (b) *d* = 310 nm, and (c) *d* = 520 nm for a width *w* = 1.5 µm. Note that three photonic lasers with thicknesses of 290 nm (*D* = 30 and 50 µm) and 310 nm (*D* = 30 µm) did not lase.

Detailed discussions about the verification of the BSW lasing

To investigate the complete BSW modes, the modes sustained by the proposed BSW laser structure have been comprehensively investigated. In the following simulation results, the BSW laser structure geometry was obtained from SEM images of the fabricated devices, which comprises five pairs of alternating TiO_2 and SiO_2 layers having respective thicknesses of 82 and 170 nm (as the BSW platform), and a R6G-doped SU-8 ridge having a thickness of 136 nm (as the top guiding structure with the oblique sidewall) lying onto the surface of the BSW platform.

Since the width of the ridge waveguide is greater than or equal to 1.5 µm, the higher-order BSW modes indeed exist in the structure and they are investigated using mode analysis. Supplementary Figures 11a–c show the calculated real parts (Supplementary Figure 11a) and imaginary parts (Supplementary Figure 11b) of the mode effective indices (expressed by $n_{\text{effective}} + ik_{\text{effective}}$) and the confinement factor (Supplementary Figure 11c) of the guided zero-order, first-order, and second-order BSW modes within the R6G-doped SU-8 ridge as a function of the width w for a thickness d of 140 nm. Both the $n_{\text{effective}}$ and the confinement factor decrease while the $k_{\text{effective}}$ increases with increasing order of the mode for the BSW mode (for w = 1.5 µm, the $k_{\text{effective}}$ of the fundamental mode increases by 5 times and 30 times for the first- and second-order modes, respectively), implying that the higher-order BSW modes possess less optical confinement and higher propagation losses. Therefore, the higher-order BSW modes are too leaky to be sustained as the lasing mode, while the fundamental BSW mode should be the natural mode for lasing in the BSW lasers.

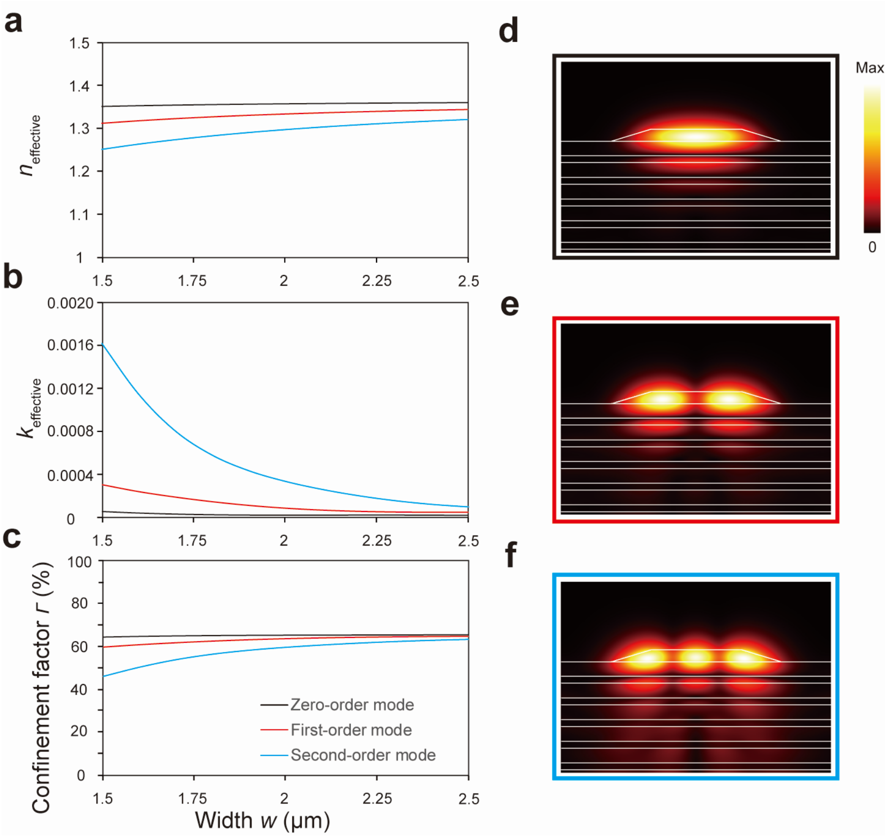

Supplementary Figure 11. Optical characteristics of the higher-order BSW modes as a function of the SU-8 polymer ridge width *w* with non-vertical sidewalls (*d* = 136 nm). (a, b) The (a) real parts and (b) imaginary parts of the mode effective indices (expressed by $n_{effective} + ik_{effective}$). (c) The confinement factor. (d–f) Calculated electric field norm distributions of the (d) zero-order, (e) first-order, and (f) second-order BSW mode for *w* = 1.5 μm. Here, the BSW platform is set to comprise five TiO_2/SiO_2 pairs having respective thicknesses of 82 nm/170 nm according to SEM images.

The internal modes are Bloch waves which exist within a periodic multilayer structure[27]. The characteristics of these modes are that their maximum electric field distributions are mainly located in the center of the multilayer (Supplementary Figure 12), implying the electric energy of such mode is confined at locations far away from the top fluorescent material (R6G-doped SU-8 polymer ridge). Thus, the coupling to internal modes is weak in the BSW laser structure. Moreover, the overlap between the electric field distribution of the internal modes and the R6G-doped SU-8 polymer (as the gain medium in this study) is also very small. Therefore, the optical feedback for such a mode is inadequate so the lasing action based on the stimulated emission of an internal mode should not be possible in BSW lasers.

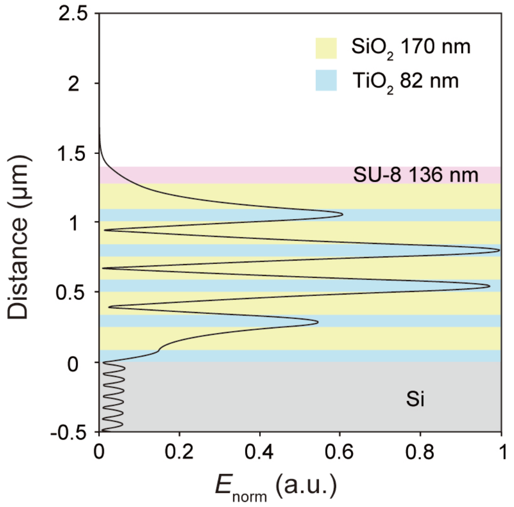

Supplementary Figure 12. The calculated one-dimensional electric field distributions for the representative internal modes of the BSW platform with a SU-8 polymer thickness of d = 136 nm.

Investigation into the BSW lasing mode by leakage radiation microscopy

The BSW lasing mode was investigated by leakage radiation microscopy[28]. The sample was firstly prepared by depositing a dielectric multilayer (BSW platform) comprising five TiO_2/SiO_2 pairs having respective thicknesses of 82 nm and 170 nm on a 100-μm-thick quartz substrate. Then, R6G-doped SU-8 polymer ring cavities were fabricated onto the surface of the BSW platform through the top-down photolithography described in the Methods section. When the sample was optically pumped by the ns pulsed laser using the conventional microscopy configuration (Supplementary Figure 7), the leakage radiation from the sample was collected simultaneously with an oil immersion objective lens having a numerical aperture (NA) of 1.4. A band-pass filter with a central wavelength of 580 nm and a full width at half maximum (FWHM) of 10 nm was placed in the optical path after the oil immersion objective lens to filter out the leakage radiation light from the stimulated process only. Then, the leakage radiation is transmitted through a tube lens and split by a 50:50 beam splitter. On one side of the beam splitter, the leakage radiation was captured by a CCD camera for optical image observation. On the other side, a convex lens was used to collect the radiation light to produce a back focal plane (BFP) image on a CCD camera (CS505MU, Thorlabs, Inc.), which is the Fourier transform of the direct plane image. By analyzing the BFP image of the BSW lasers, the wave-vector information (e.g., the effective refractive index) of the lasing mode can then be obtained.

Supplementary Figure 13 shows the BFP images of the BSW laser having a diameter D of 100 μm (d = 136 nm and w = 2.5 μm) obtained below (Supplementary Figure 13a) and above (Supplementary Figure 13b) the lasing threshold. Several rings are observed, and each ring corresponds to a leakage radiation mode from the BSW laser. The effective refractive index ($n_{\text{effective}}$) of the mode can be expressed by NA in the BFP image and is derived by the relation:

$$n_{\text{effective}} = \text{NA in the BFP image} = 1.4 \times \frac{R_{\text{mode}}}{R_{\text{edge}}}, \text{(S3)}$$

where R_{mode} is the radius of the ring, Redge the radius of the edge of the BFP image, which

corresponds to the NA of the oil immersion objective lens used in the setup (NA = 1.4). In Supplementary Figure 13a, the inner ring (NA = 1.06) corresponds to the BSW mode sustained by the bare multilayer (without top R6G-doped SU-8 polymer ridge), and the outer rings should correspond to multilayer modes, including the internal modes and the BSW-guided modes within the SU-8 ridge waveguide. When the pump energy density increases above the lasing threshold of the BSW laser (Supplementary Figure 13b), all of the rings in the BFP image become brighter, and a very bright ring is observed with NA ~1.32 to 1.4, which can be attributed to the leakage radiation of the lasing emissions. Note that the NA value of the lasing emission agrees well with the calculated $n_{effective}$ of the BSW mode ($n_{effective}$ = 1.36, as seen in Supplementary Figure 15c). According to these results, the lasing mode of the BSW lasers is confirmed to be indeed the BSW mode.

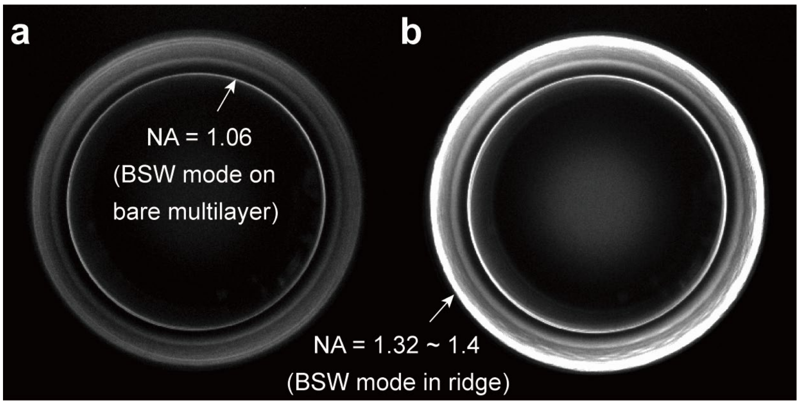

Supplementary Figure 13. BFP images of the BSW laser having a diameter *D* of 100 μm (*d* = 136 nm and *w* = 2.5 μm) obtained (a) below and (b) above the lasing threshold using leakage radiation microscopy.

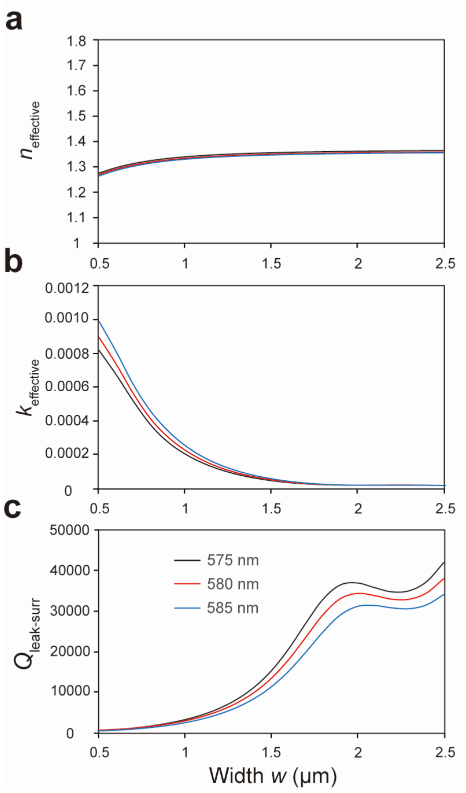

Supplementary Figure 14. The analysis of the $Q_{leak-surr}$ of the BSW ring cavities at different wavelengths. The (a) real part and (b) imaginary part of the mode effective indices at wavelengths of 575 (black), 580 (red), and 585 nm (blue). (c) The simulated surrounding losses (defined as $Q_{leak-surr}$) of the BSW modes using mode analysis (diameter independent results). The BSW platform comprises five TiO_2/SiO_2 pairs having respective thicknesses of 82 nm/170 nm. Although the $Q_{leak-surr}$ of the BSW ring cavities exhibits weak wavelength dependence (lower propagation loss for shorter wavelength due to higher optical confinement), a strong dependency with the ridge width is observed.

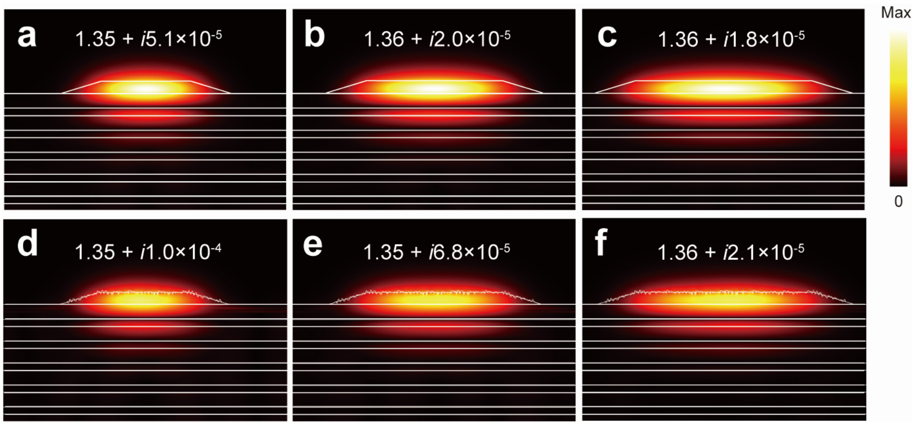

Supplementary Figure 15. Effect of surface roughness on the mode effective index for different ridge widths. (a–c) The calculated electric field norm distributions of the BSW modes (d = 136 nm) for (a) w = 1.5 μm, (b) w = 2 μm, and (c) w = 2.5 μm. (d–f) The calculated electric field norm distributions of the BSW modes (d = 136 nm) for (d) w = 1.5 μm, (e) w = 2 μm, and (f) w = 2.5 μm when surface roughness was introduced (root-mean-square roughness was set to 10 nm, a value corresponding to a tradeoff between computing time and mesh size). The BSW platform comprises five TiO_2/SiO_2 pairs having respective thicknesses of 82 nm/170 nm. The real and imaginary parts of the mode effective indices (expressed by $n_{effective} + ik_{effective}$) are indicated in the figure. The simulated increase in $k_{effective}$ due to roughness are 5.1×10^{-5}, 4.8×10^{-5}, and 3×10^{-6} for the ridge widths w of 1.5, 2, and 2.5 μm, respectively. The increased losses are attributed to scattering, and the scattering losses become larger with decreasing width of the ridge.

Detailed discussions about the Q_{leak} of the BSW ring cavities

The Q_{leak} of the BSW ring cavities for the ridge with non-vertical sidewalls as a function of width for different diameters were simulated, using eigenfrequency analysis for an axisymmetric model, and compared the results with the $Q_{leak-surr}$, as displayed in Supplementary Figures 16a–d. The Q_{leak} values are found to be independent of the ridge width for the BSW ring cavities having large widths. Once the ridge width decreases such that the value of $Q_{leak-surr}$ is reduced to be comparable to that of the Q_{leak}, Q_{leak} starts to decrease with a similar trend than that of the decrease in the $Q_{leak-surr}$ for small ridge widths. This behavior can be understood from the simulated electric field norm distributions for the WGM resonances of the BSW ring cavities having various diameters and ridge widths, as shown in Supplementary Figures 16e–h. For the case that the ridge width is large enough (i.e., $w = 2.5$ μm), the WGM modes in the BSW ring cavities are readily supported by the ring cavity. Note that these distributions of the WGM modes are pushed outward in the radial direction, and the distribution becomes smaller with decreasing diameter due to the effect of the curvature. When the ridge width further decreases from $w = 2.5$ μm, the WGM modes are still supported by the ring cavity and the distributions remain the same. In this case, the Q_{leak} is independent of the ridge width, suggesting that the Q_{leak} is evaluated by only considering the WGM mode distribution in the eigenfrequency analysis. When the ridge width is decreased until the inward ridge edge becomes close to the WGM mode distribution, then the $Q_{leak-surr}$ becomes comparable to the Q_{leak}. A further decrease in the ridge width results in a mode distribution with an electric field distribution similar to that of a guided BSW mode within a ridge waveguide (see Supplementary Figures 16i and 16j), and the Q_{leak} is found to be similar to the $Q_{leak-surr}$ for small ridge widths. According to these results, we conclude that the simulated Q_{leak} using eigenfrequency analysis for an axisymmetric model corresponds to the $Q_{leak-surr}$ only when the mode distributions are the same, that is, the WGM mode distributions as obtained by eigenfrequency analysis become similar to the distributions for the mode analysis.

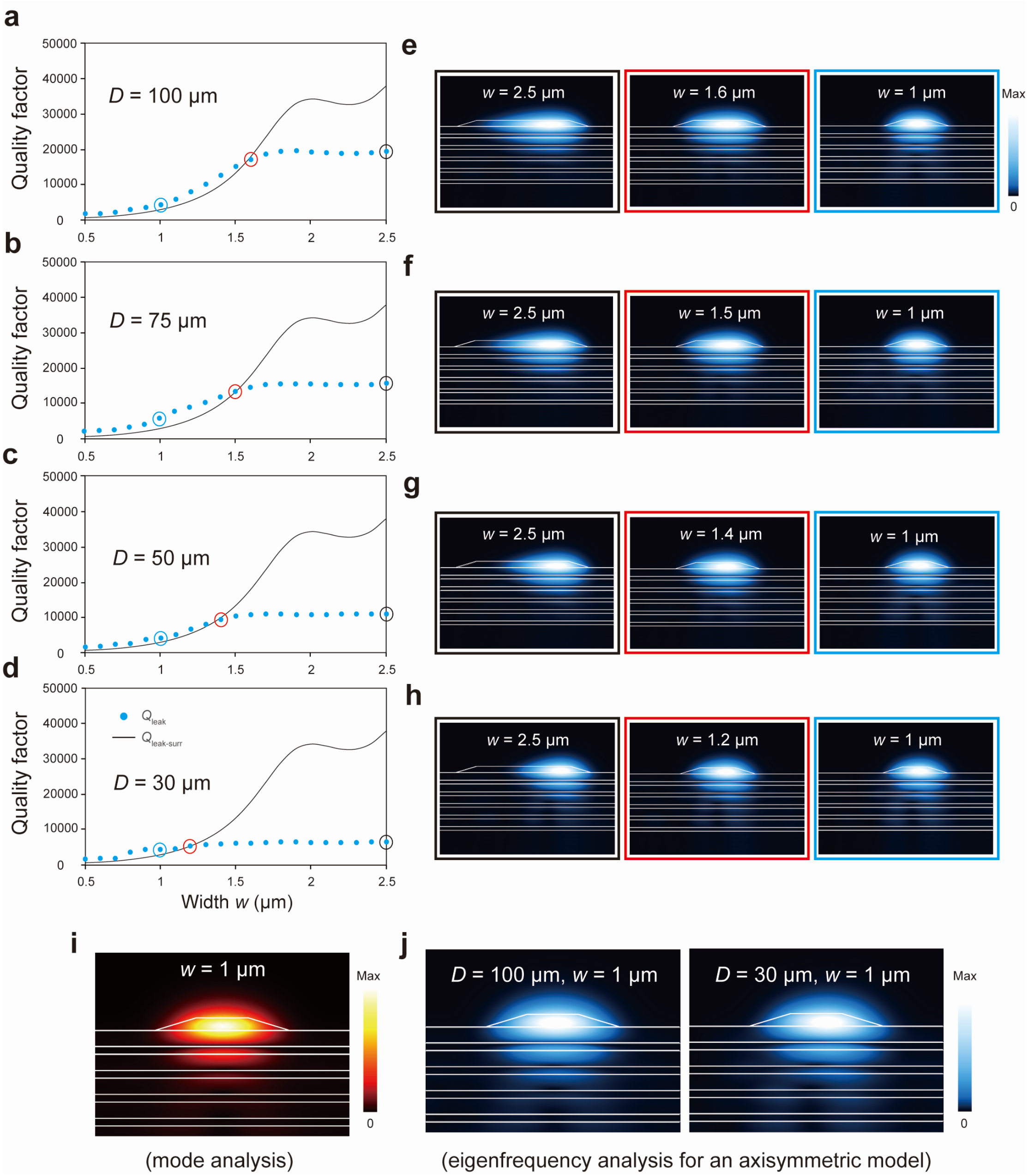

Supplementary Figure 16. The detailed investigation of the Q_{leak} of the BSW ring cavities with various diameters and ridge widths. The BSW platform comprises five TiO_2/SiO_2 pairs having respective thicknesses of 82 nm/170 nm. (a–d) The comparisons between the simulated $Q_{\text{leak-surr}}$ of the BSW modes using mode analysis (diameter independent results) and the simulated Q_{leak} of the ring laser cavities having diameters of (a) D = 100 μm, (b) D = 75 μm, (c) D = 50 μm, and (d) D = 30 μm obtained by eigenfrequency analysis for an axisymmetric model. (e–h) The calculated electric field norm distributions for the BSW ring laser cavities with (e) D = 100 μm, (f) D = 75 μm, (g) D

= 50 μm, and (h) D = 30 μm obtained by eigenfrequency analysis for an axisymmetric model. Left: the distributions for the ridge width w = 2.5 μm; middle: the distributions for ridge width at which the $Q_{\text{leak-surr}}$ is approximately equal to Q_{leak}; right: the distributions for the ridge width w = 1 μm. (i) The calculated electric field norm distribution of the BSW mode for d = 136 nm and w = 1 μm using mode analysis. (j) The calculated electric field norm distributions of the ring laser cavities (d = 136 nm, w = 1 μm) for D = 100 μm (left) and D = 30 μm (right), respectively, using eigenfrequency analysis for an axisymmetric model.

From the above investigation, it is concluded that Q_{leak} and $Q_{\text{leak-surr}}$ give similar results when the BSW ring cavities possess the same distribution as simulated in an axisymmetric model (eigenfrequency analysis) and straight waveguide model (mode analysis). Thus, we assumed that the lateral radiation leakage due to curvature should be almost zero ($1/Q_{\text{leak-curv}} \approx 0$). To verify this assumption, a simulation model combined with effective index method (EIM) and two-dimensional (2D) eigenfrequency analysis (COMSOL software) is employed to simulate the $Q_{\text{leak-curv}}$[29], as displayed in Supplementary Figures 17a–c. In this model, a 2D structure of a BSW ring cavity is divided into two regions, including the BSW platform without (bare, blue) and with (ridge, red) a 136-nm-thick top SU-8 polymer ridge (Supplementary Figure 17a). The effective indices of these two regions are calculated using the mode analysis, as displayed in Supplementary Figure 17b. Note that the lateral radiation leakage due to the effect of curvature is only related to the effective refractive index ($n_{\text{effective}}$) difference between such two regions. It is also noted that the effect of the oblique sidewall has been taken into account in the mode analysis (regarding the Q_{leak} investigation for the SU-8 polymer ridge with vertical sidewalls, see Supplementary Figures 17d–f). Supplementary Figure 17c shows the simulated $Q_{\text{leak-curv}}$ of the BSW ring cavities (d = 136 nm, w = 1.5 and 2.5 μm) for different diameters. The $Q_{\text{leak-curv}}$ values of the BSW ring cavities are relatively high ($Q_{\text{leak-curv}} \geq 10^{11}$), suggesting that the lateral radiation leakage due to curvature can be neglected.

Based on these investigations, the light leakage to the surrounding is indeed a dominant loss for our proposed BSW ring cavities.

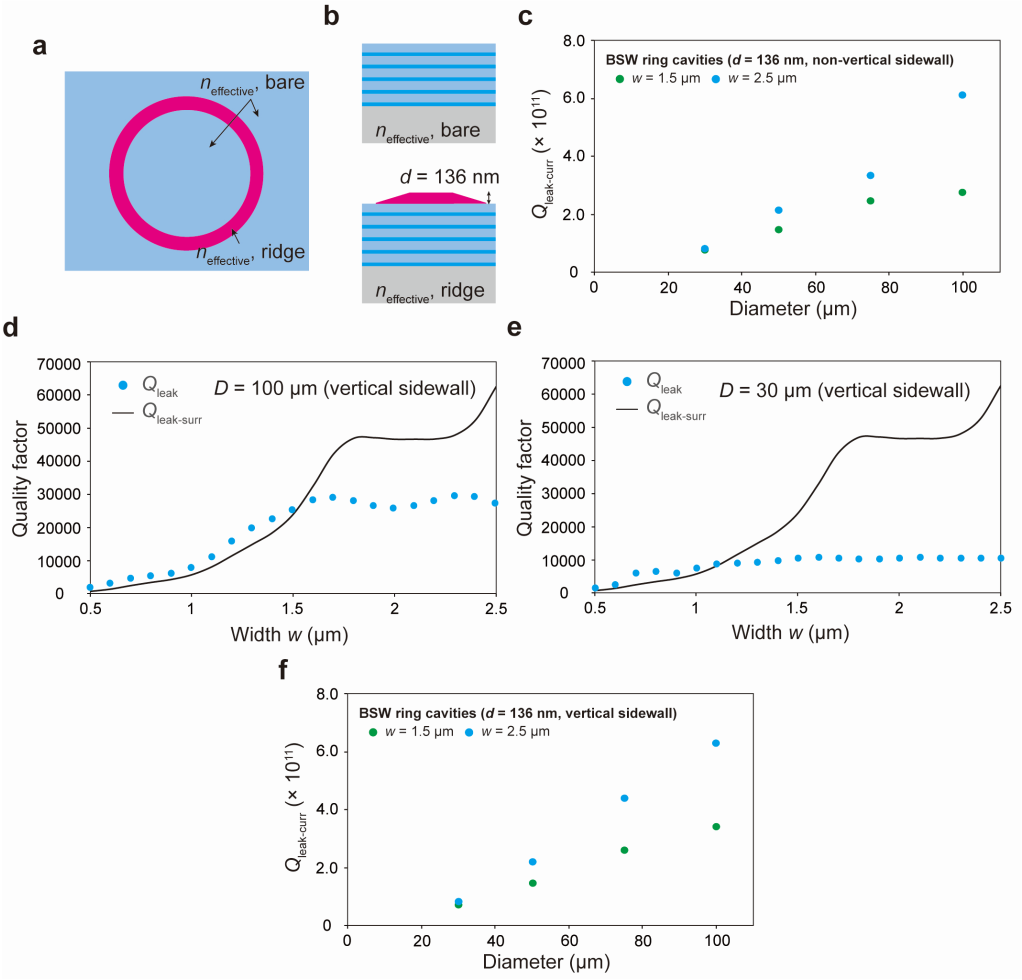

Supplementary Figure 17. Investigation into the $Q_{\text{leak-curv}}$ of the BSW laser ring cavities having various diameters. (a, b) Schematic representations of the 2D eigenfrequency analysis for simulating the $Q_{\text{leak-curv}}$: (a) top view and (b) cross-sectional view of the simulation model for calculating the effective indices for bare (blue in figure a) and ridge (red in figure a) regions. The BSW platform comprises five TiO_2/SiO_2 pairs having respective thicknesses of 82 nm/170 nm. (c) The simulated $Q_{\text{leak-curv}}$ of the BSW ring laser cavities (d = 136 nm, w = 1.5 and 2.5 μm) having various diameters. (d, e) The comparisons between the simulated $Q_{\text{leak-surr}}$ of the BSW modes using mode analysis (diameter independent results) and the simulated Q_{leak} of the ring laser cavities having diameters of (d) D = 100 μm and (e) D = 30 μm obtained by eigenfrequency analysis for an axisymmetric model.

Here, the sidewall of the SU-8 polymer ridge width is vertical. (f) The simulated $Q_{leak-curv}$ of the BSW ring laser cavities (d = 136 nm, w = 1.5 and 2.5 μm with vertical sidewall) having various diameters.

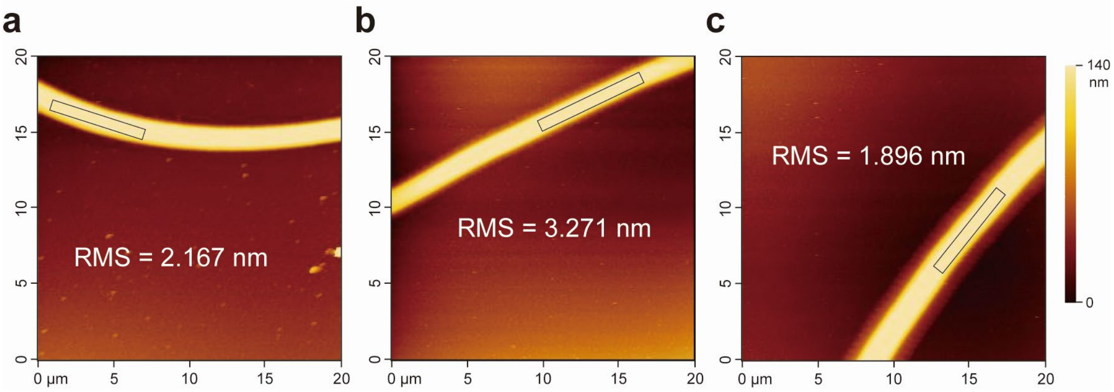

Supplementary Figure 18. AFM images of the R6G-doped SU-8 ridge of the ring cavity onto the surface of the BSW platform with the corresponding root means square (RMS) roughness: (a) ridge width $w = 1.5$ µm, (b) $w = 2$ µm, and (c) $w = 2.5$ µm. RMS roughness was calculated for the indicated areas.

Investigation of multilayer design for the BSW laser

From the dispersion diagram of the BSW mode (Supplementary Figure 2), one can assume that the BSW mode wavevector will become larger when designing the multilayer for a red-shifted forbidden band. Also, a BSW mode in the lasing band could still be sustained within a SU-8 polymer ridge waveguide having a smaller thickness by increasing the degree of confinement of the BSW mode. To investigate this behavior, we calculated the BSW dispersion diagram of the TiO_2/SiO_2 multilayer having the respective thicknesses of 71 nm/154 nm (original design) for a SU-8 layer on the multilayer top including the case of a smaller thickness of 100 nm (Supplementary Figure 19a). For comparison purposes, the BSW dispersion diagrams for the red-shifted forbidden band with different TiO_2/SiO_2 multilayers (two additional designs with thicknesses of 78 nm/170 nm and 89 nm/194 nm, which are designed at wavelengths of 640 and 730 nm, respectively, based on the equation S1) having a top 100-nm-thick SU-8 layer were also computed (Supplementary Figure 19b). In Supplementary Figure 19a, a smaller thickness of the SU-8 polymer layer results in less optical confinement (as displayed in Figures 1a–c), and the dispersion diagram exhibits smaller wavevectors k_x. As shown in Supplementary Figure 19b, when the forbidden band of the TiO_2/SiO_2 multilayer redshifts, the dispersion of BSW modes red-shift simultaneously, implying larger wavevectors k_x for the BSW modes in the lasing band (at approximately 580 nm) of the R6G-doped SU-8 ring lasers (see inset of Supplementary Figure 19b). To further characterize the optical properties of these BSW modes, we calculated (Supplementary Figures 19c–e) using mode analysis the mode effective indices, the confinement factors, and the electric field norm distributions of these BSW modes sustained within the SU-8 polymer ridge (d = 100 nm, w = 2.5 μm) lying onto the surface of different TiO_2/SiO_2 multilayers. It is found that, when red-shifting the forbidden band of the designed multilayer, the $n_{effective}$ increases while the $k_{effective}$ decreases, suggesting a better-supported BSW mode as expected from the dispersion diagram. However, the confinement factor (defined as the ratio between the electric energy in the SU-8 ridge and the total electric energy of

the mode) decreases when the forbidden band of the designed multilayer was red-shifted. We attribute this behavior to a broadening of the fields in the top SiO_2 layer underneath the SU-8 polymer ridge (see Supplementary Figures 19c–e), which will likely result in decreased optical feedback provided for the WGM resonance, especially for small ring diameters resulting in a significant curvature effect.

To clarify possible improvement in the BSW laser design, we investigated the quality factors of the BSW ring cavities (d = 100 nm, w = 2.5 μm) for very large diameters by using 2D guiding structures (for which the curvature effect is neglected), and a relatively small diameter of D = 30 μm onto the surface of different BSW platforms. For the case of very large ring diameters, the effect of the curvature can be neglected so that the leakage-related quality factor Q_{leak} can be estimated by only considering the quality factor $Q_{\text{leak-surr}}$, which is related to the leakage of light to the surrounding (Supplementary Figure 19f). For the small diameter, the effect of curvature should be taken into account so that the quality factor (Q_{leak}) is evaluated using the eigenfrequency analysis for an axisymmetry model (Supplementary Figure 19g). As expected, the Q_{leak} values for the BSW ring cavities with large diameters increase with red-shifting the forbidden band of the designed multilayer due to smaller $k_{\text{effective}}$. However, the Q_{leak} values for the BSW ring cavities with a small diameter show a maximum when the BSW platform consists of five TiO_2/SiO_2 pairs having respective thicknesses of 78 nm/170 nm, which is attributed to a tradeoff between the decrease in $k_{\text{effective}}$ and confinement factor. In summary, the quality factor of the BSW ring cavity can indeed be improved by red-shifting the forbidden band of the multilayer, thus suggesting an improved lasing performance even when the thickness of the SU-8 ridge becomes small.

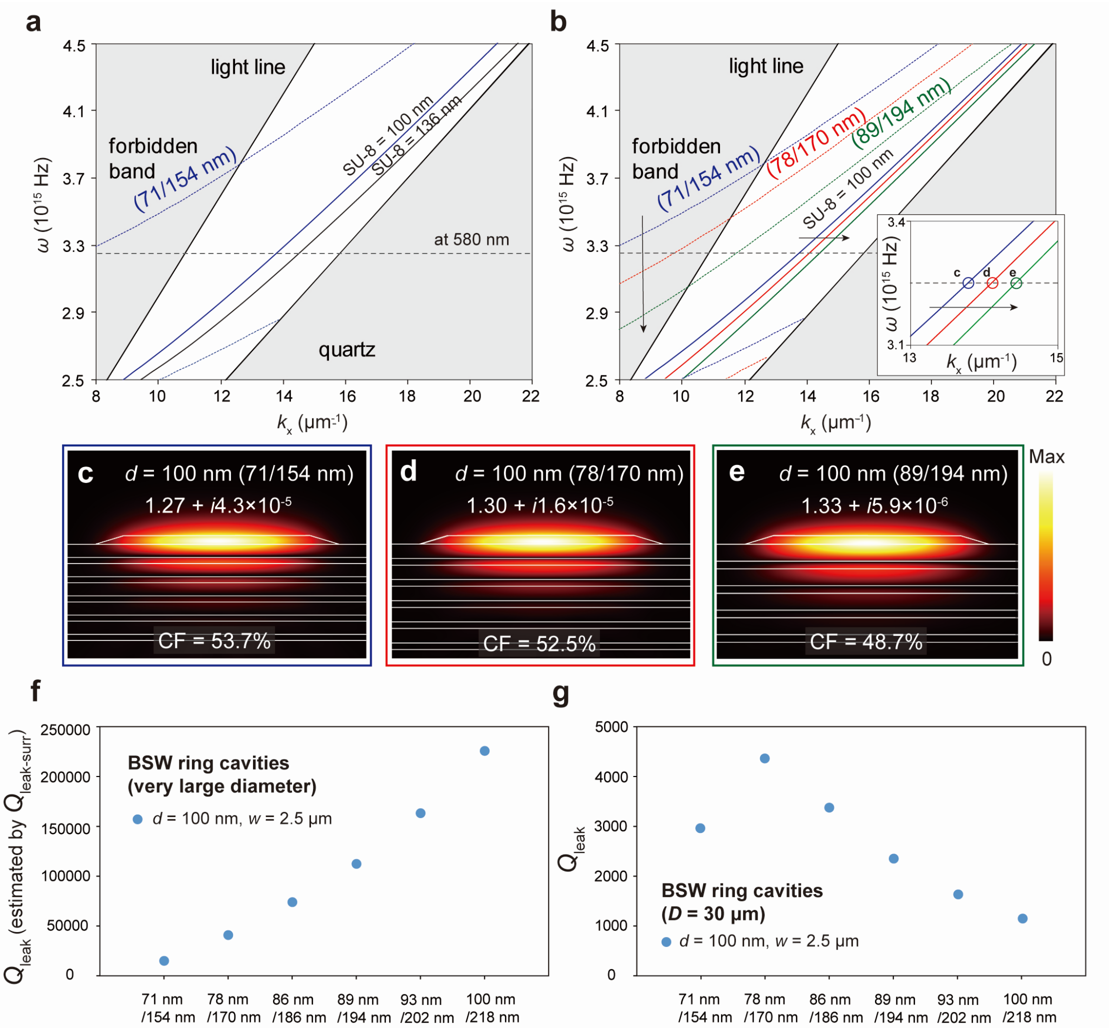

Supplementary Figure 19. Investigation into the multilayer design of the BSW laser with a smaller thickness of the SU-8 polymer layer. (a) The BSW dispersion diagrams of the TiO_2/SiO_2 multilayer, having respective thicknesses of 71 and 154 nm with a 136-nm-thick (black) and a 100-nm-thick (blue) top SU-8 polymer layer. (b) The BSW dispersion diagrams of the TiO_2/SiO_2 multilayers having respective thicknesses of 71 nm/154 nm (blue), 78 nm/170 nm (red), and 89 nm/194 nm (green) with a top 100-nm-thick SU-8 layer. The forbidden bands of the TiO_2/SiO_2 multilayers are displayed, and the interested wavelength (580 nm) corresponding to the center of the lasing band in the R6G-doped SU-8 ring lasers is also indicated. Inset in figure b: The magnified graph of the dispersion diagrams showing larger wavevector k_x when designing the multilayer for a red-shifted forbidden band. (c–e) The calculated electric field norm distributions of the BSW modes sustained within the SU-8 polymer ridge (d = 100 nm, w = 2.5 μm with non-vertical sidewall) onto the surface

of TiO_2/SiO_2 multilayers having respective thicknesses of (c) 71 nm/154 nm, (d) 78 nm/170 nm, and (e) 89 nm/194 nm using mode analysis. The real and imaginary part of the mode effective indices (expressed by $n_{\text{effective}} + ik_{\text{effective}}$) and confinement factor (CF) are indicated in the figure. (f, g) The simulated Q_{leak} of the BSW ring cavities (d = 100 nm, w = 2.5 μm) for (f) very large diameters (for which the curvature effect can be neglected) and (g) a small diameter of D = 30 μm onto the surface of different multilayer using the eigenfrequency analysis for an axisymmetry model.

In the following, the above strategy was applied to fabricate BSW lasers with improved performance. The effect of the multilayer design was investigated on the BSW ring lasers with a thickness of 136 nm and improved performance in terms of the lasing threshold for small ring diameters was demonstrated. Supplementary Figure 20a shows the calculated BSW dispersion diagram of the TiO_2/SiO_2 multilayers having respective thicknesses of 71 nm/154 nm (original design) and 89 nm/194 nm, both with a SU-8 layer thickness of 136 nm. As seen in the previous section, the wavevector k_x for the BSW modes increases with red-shifting the forbidden band of the multilayer, and an increase in $n_{\text{effective}}$ and a decrease in $k_{\text{effective}}$ are observed (see Supplementary Figures 20b, c). Note that the value of the confinement factors slightly decreases, indicating that this multilayer design should benefit the BSW ring lasers even for small ring diameters.

To clarify the possible improvement, the Q_{leak} of the BSW ring cavities (d = 136 nm, w = 2.5 μm, D = 30 μm) onto the surface of multilayers designed with different forbidden bands were calculated using the eigenfrequency analysis for an axisymmetric model, as shown in Supplementary Figure 20d. The Q_{leak} value is found to be optimized (Q_{leak} ~16,000) when the multilayer consists of five TiO_2/SiO_2 pairs having respective thicknesses of 89 nm/194 nm. Next, the BSW ring cavities having diameters D in the range between 10 to 100 μm (thickness d = 136 nm, width w = 2.5 μm) were fabricated onto the surface of the improved BSW platform (consisting of five pairs of TiO_2 and SiO_2 layers having respective thicknesses of 89 nm/194 nm). Then the

fabricated BSW lasers were optically pumped with a nanosecond laser to examine their lasing performances (Supplementary Figure 20e). The measured lasing threshold values of the BSW lasers having the large diameters of 50, 75, and 100 μm are approximately the same as the BSW lasers onto the original design multilayer (Figure 5a), implying that the lasing thresholds of the revised design should be limited by other losses than the leakage-related losses (losses from the effect of curvature and propagation) such as the surface scattering losses. Importantly, it is found that the lasing threshold value is significantly reduced to 33% of that of the original design for the BSW laser with a diameter of 30 μm, which is attributed to the decrease in leakage-related losses. Furthermore, the minimum diameter achieving lasing is shrunk to 20 μm (Supplementary Figure 20e). According to these results, we confirmed that the lasing performance of the BSW lasers could be improved using a better design of the BSW platform.

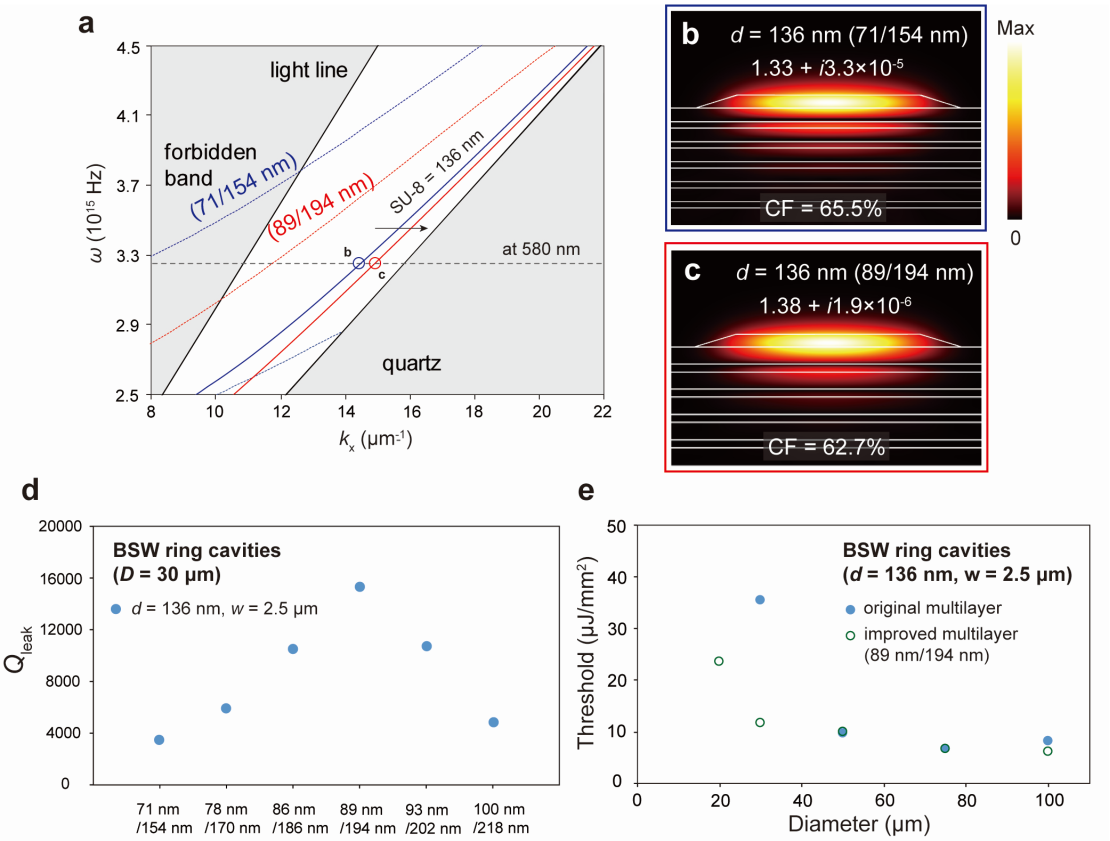

Supplementary Figure 20. Improved lasing performance for the BSW lasers in terms of the multilayer design. (a) The BSW dispersion diagrams of the TiO_2/SiO_2 multilayers having respective

thicknesses of 71 nm/154 nm (blue) and 89 nm/194 nm (red), both with a 136-nm-thick top SU-8 polymer layer. (b, c) The calculated electric field norm distributions of the BSW modes sustained within the SU-8 polymer ridge (d = 136 nm, w = 2.5 μm with non-vertical sidewall) onto the surface of TiO_2/SiO_2 multilayers having respective thicknesses of (b) 71 nm/154 nm and (c) 89 nm/194 nm. The real and imaginary part of the mode effective indices (expressed by $n_{effective} + ik_{effective}$) and confinement factor (CF) are indicated in the figure. (d) The simulated Q_{leak} of the BSW ring cavities (d = 136 nm, w = 2.5 μm, D = 30 μm) onto the surface of different multilayer using the eigenfrequency analysis for an axisymmetry model. (e) The measured lasing threshold values of the BSW ring lasers (d = 136 nm, w = 2.5 μm) onto the surface of the original (blue circle) and improved (green ring) BSW platforms.

Coupling between a BSW ring laser cavity and a waveguide

To demonstrate the possibility of using the BSW laser for optical integrated circuits, we have investigated the coupling between a BSW ring laser cavity and a waveguide using the finite-difference time-domain (FDTD) technique (COMSOL software). As displayed in Supplementary Figures 21a and 21b, the simulation model is set to be composed of a curved waveguide (a fraction of the BSW ring laser cavity having a diameter of 30 μm) and a straight waveguide, and the smallest distance between them is defined as the coupling gap. A BSW mode is launched from one end of the input curved ridge waveguide and propagates along the tangential-direction. Some portion of the guiding BSW mode will couple to the adjacent waveguide and then propagates to the end of the output ridge waveguide. Then, the coupling efficiency is estimated as the ratio between the power at the output ridge and input ridge (P_{out}/P_{in}). Supplementary Figure 21c shows the simulated electric field norm distribution for the coupling between the SU-8 polymer ring cavity and the ridge waveguide, and the coupling efficiency for the BSW laser as a function of the gap is reported in Supplementary Figure 21d. The coupling efficiency increases with decreasing the gap, and a value of approximately 0.2% is obtained for a gap of 0.050 μm. Note that these results were almost the same as those obtained by lunching the BSW mode in the guiding slab and coupling to the SU-8 polymer ring cavity. In addition, the estimated coupling efficiencies are of the same order than that of a structure realizing standard evanescent coupling[30], thus suggesting the validity of our simulation model. Although the estimated coupling efficiency is relatively low, we believe that more efficient couplings between a BSW ring laser and a BSW waveguide can be obtained via improved coupling designs such as wrapped waveguide[31]. Accordingly, the applicability of the proposed BSW lasers in integrated optical circuits is established so that future applications of BSW lasers on a chip such as optical modulators and logic devices can be realized.

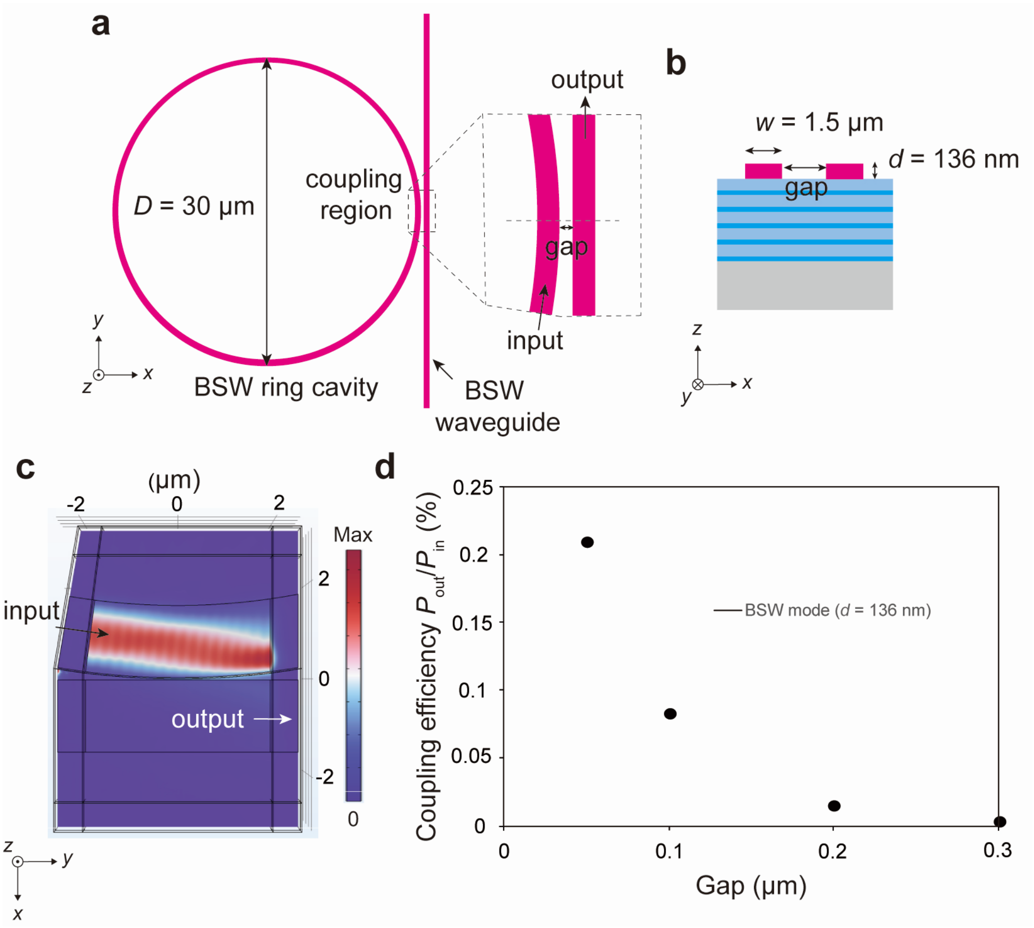

Supplementary Figure 21. Investigation into the coupling between a BSW ring laser cavity and a BSW guiding slab. (a, b) Schematic representation of the (a) coupling between a BSW ring laser cavity and a straight BSW waveguide, and (b) cross-sectional view of the simulation model. The width of the SU-8 polymer ridge is 1.5 μm. (c, d) The simulated (c) electric field norm distribution for coupling between the BSW ring laser cavity and the waveguide and (d) the estimated coupling efficiency as a function of the gap.

Photobleaching of the BSW lasers made of R6G-doped SU-8 polymer

The photobleaching of the BSW lasers is investigated by continuously pumping laser devices and collecting their emission intensities. Photobleaching is a common photo-induced degradation for dye molecules. When exposed to intense light, the local heating near the dye molecules during pumping enables them to receive mobility and then react with the polymer matrix, the impurities (e.g., oxygen), or the other dye molecules, resulting in degradation of the fluorescent dyes[19]. Supplementary Figure 22 shows the normalized emission intensity of a BSW laser (D = 100 μm) as a function of the number of pump pulses. The emission intensity decreases to half of its initial value after approximately 20,000 pulses. Note that this lifetime is of a similar order of magnitude as that reported for R6G-doped SU-8 polymer lasers[19,32]. Although the photobleaching of dye-doped polymer restricts the lifetime of the proposed BSW laser, the fabrication method is simple, cheap, and rapid, making it suitable for large production. Furthermore, the photobleaching can be reduced by operating the laser at low temperature[33], working under an inert atmosphere[33] , or filling the free volume in the polymer host with additives (such as diphenyl thiourea[34]) could improve the photostability of such BSW lasers and make them more favorable in long-term applications. It is also noted that the R6G-doped SU-8 polymer was chosen as the gain medium in this study to provide a proof of concept for the BSW laser. We believe that the applicability of the proposed BSW laser can be drastically extended by employing an inorganic gain medium (e.g., Er-doped Si and GaN) so that the biosensing applications of the BSW lasers will be made possible.

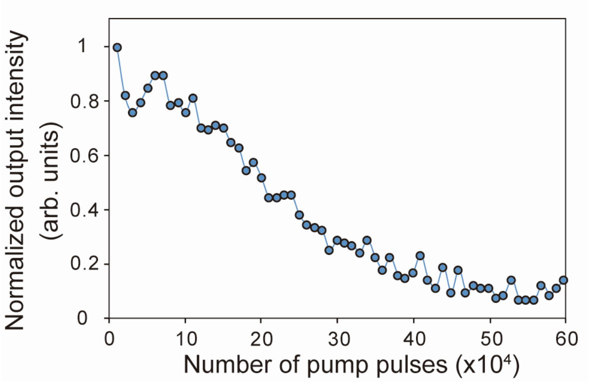

Supplementary Figure 22. The normalized emission intensity of the BSW laser as a function of the number of pump pulses. The diameter of the BSW laser was 100 μm and the pump energy density 10.9 μJ/mm^2.

Environmental sensitivity investigation of the BSW lasers

Before investigating the environmental sensitivity of the BSW lasers, we first investigated the interaction between the SU-8 polymer and toluene vapor using a quartz crystal microbalance (QCM) [35] detection system to clarify the sensing mechanism for the SU-8 polymer. The well-known detection mechanism of QCMs relies on the shift of the resonance frequency of a quartz crystal resonator (QCR) upon deposition of a thin film on one of the sides of the quartz crystal. Transversal oscillation modes (e.g., breathing modes) are used to reduce interaction with the atmosphere just above the quartz crystal and thus realize a high-quality factor of resonance that enables high sensitivity to the thickness of deposited layers onto the quartz crystal.

An R6G-doped SU-8 polymer layer was deposited onto the surface of a QCR with a nominal resonance frequency of 5 MHz (CRTM-1000, ULVAC). To ensure that the properties of the SU-8 polymer layer are nearly identical to those of the BSW lasers, the same fabrication process was employed as for the BSW lasers, except for the presence of the photomask. Also, a bare QCR without a SU-8 polymer layer was prepared for comparison purposes. The QCRs were then subjected to toluene vapor by inserting them into a beaker containing liquid drops of toluene (the QCRs are not immersed in toluene and the beaker is sealed to reach saturated toluene vapor) and their resonance frequencies were monitored and converted to a change in thickness using the parameters for a carbon layer. Supplementary Figure 23a displays the observed change in the thickness of the QCRs without and with the SU-8 polymer layer when exposed to toluene vapor. In the presence of toluene vapor, the estimated thickness of the SU-8 polymer-coated QCR increased to approximately 35 Å within 300 s. In contrast, the thickness on the bare QCM remained the same. This result confirms that the SU-8 polymer interacts with toluene vapor. This interaction shown in Supplementary Figure 23a could be related to the condensation and sorption of toluene on the surface and/or diffusion of toluene molecules into the SU-8 polymer (swelling). Because a change in the thickness is not observed on the bare QCR (negligible condensation and sorption on the metal

surface of the bare QCR) and the relatively large change in the estimated thickness of the SU-8 polymer recovers (complete recovery is not reached due to the limited testing time) when the SU-8 polymer-coated QCR is removed from the toluene vapor, we infer that both sorption of toluene vapor and sorption followed by swelling should be the possible contributions when SU-8 polymer is exposed to toluene vapor.

To fully rule out any effect of condensation on the SU-8 polymer, the QCM system was connected to a water chiller that was used to control the temperature of the QCRs (the temperature was set to 12°C). The temperature-controlled QCRs were then exposed to toluene vapor to observe the condensation of toluene gas molecules onto the surface of the QCRs, as shown in Supplementary Figure 23b. Here, the experiment was conducted in a glove box with low humidity (relative humidity of 20%) to avoid the condensation of the water vapor. In Supplementary Figure 23b, the QCR sample with the SU-8 polymer layer shows a response to toluene vapor at the initial stage (room temperature). After turning on the cooling system, the estimated thickness of the QCR with the SU-8 polymer layer still increases at a constant rate and saturates to approximately 10 Å in 350 s, and then a sudden increase occurs at about 630 s. Because this sudden increase was not observed for the QCR with the SU-8 polymer layer in the absence of toluene vapor at a low temperature (see inset of Supplementary Figure 23b), we attribute this response to the condensation of the toluene gas molecules (we could also confirm condensation of toluene on the bare QCR). It is noted that the low increase rate at the initial stage should correspond to the sorption of toluene vapor or sorption followed by swelling of the SU-8 polymer. According to these results, we could confirm that the sensing mechanism of the proposed BSW lasers should be related to the effect of sorption or sorption followed by swelling of the SU-8 polymer ridge.

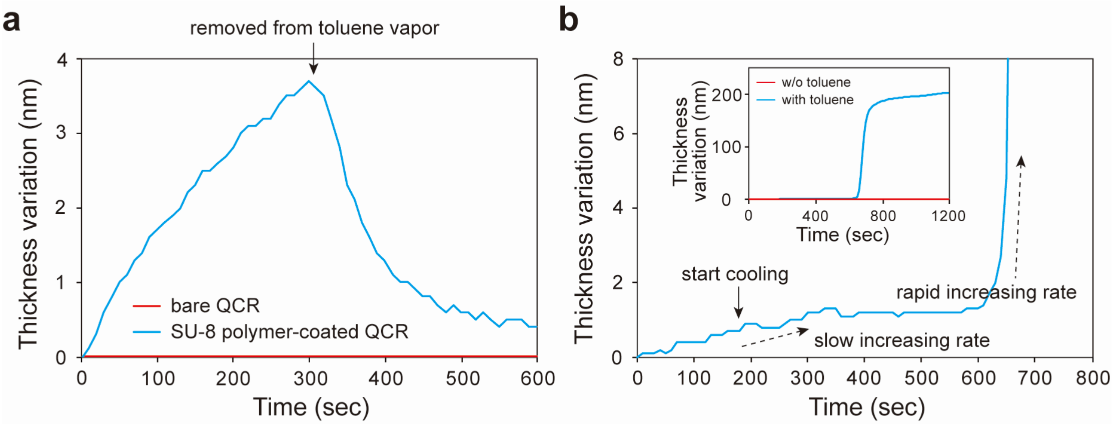

Supplementary Figure 23. The change in the thicknesses of QCRs without (bare QCR in red) and with a SU-8 polymer coating (SU-8 polymer coated QCR in blue) in response to toluene vapor. (a) Responses to toluene and recoveries at room temperature. (b) Response to toluene when the temperature of the QCR is controlled (note the slow increase rate attributed to sorption of toluene vapor or sorption followed by swelling, and the fast rate attributed to condensation). Inset: the full response of QCRs with a SU-8 polymer coating in the absence (red) and presence (blue) of toluene vapor.

The environmental sensitivity of the proposed BSW lasers is then investigated by monitoring the lasing wavelength in the presence of toluene vapor. The experimental details are described as follows. First, the BSW laser sample was placed inside a chamber connected to a cylinder with its gas valve closed. A droplet of 50 μL toluene was added inside that cylinder beforehand. After optically pumping the BSW laser sample, the system was maintained with the valve closed for 60 min to obtain saturated vapor of toluene, and then the BSW laser sample was optically pumped again to check the stability of the structure. Next, the gas valve was opened so that the saturated vapor of toluene started to diffuse into the chamber. Once the toluene molecules reached the BSW laser, they could just sorb onto the surface or sorb and then diffuse into the R6G-doped SU-8 polymer ridge and thereby swelled the structure. Then, the emission spectra of the BSW laser as a function of time were collected for studying the sensitivity of the BSW lasers (Figure 6b).

Because the sensing mechanism of the proposed BSW lasers should be related to the sorption of toluene vapor or sorption followed by swelling of the SU-8 polymer ridge, both the sorption and the swelling effect are theoretically investigated. For the sorption effect, it is assumed that a toluene layer (n = 1.50) is deposited onto the surface of the SU-8 polymer ridge. On the other hand, for the swelling effect, the increase in both the ridge size and refractive index of the SU-8 polymer ridge is assumed. Supplementary Figure 24a displays the simulated wavelength shifts of the BSW ring lasers with an additionally deposited toluene layer (n = 1.50, considering only the sorption of toluene vapor) onto the surface of the SU-8 polymer ridge. For comparison purposes, the simulated wavelength shifts of the photonic laser (d = 520 nm) with a toluene layer are also displayed (Supplementary Figure 24b). Supplementary Figures 24c,d display the simulated wavelength shifts of the BSW ring lasers with respect to the increase in the ridge size (with constant refractive index) and the increase in the refractive index of SU-8 polymer ridge (with constant ridge size), respectively. Again for comparison purposes, the simulated wavelength shifts of the photonic laser (d = 520 nm) with respect to the increase in the ridge size (with constant refractive index) are also displayed (Supplementary Figure 24e). In these investigations, the eigenfrequency analysis for an axisymmetric model was utilized. It is found that when the toluene layer is deposited onto the surface (sorption) and the ridge size or refractive index of the SU-8 polymer increases (swelling), the resonant wavelength of the BSW ring laser red-shifts linearly. Because the refractive index of the deposited toluene layer is close to that of the SU-8 polymer, both the sorption and swelling effect (considering the increase in the ridge size Δa, as displayed in Supplementary Figure 24c) exhibit similar wavelength shifts for the BSW ring lasers. The deposition of the toluene layer of 0.14 nm (Supplementary Figure 24a) and the increase in the ridge size of 0.1 nm (Supplementary Figure 24c) or a change in the refractive index of 0.00023 (Supplementary Figure 24d) corresponds to a 0.05 nm wavelength shift, which is of the same order as the observed wavelength shift in Figure 6b. Regarding the investigation of the swelling effect, it should be noted that the simulated values for the changes that can explain the lasing shifts are of the same order than those measured for the

swelling of non-baked SU-8 polymer[36]. It is also worth mentioning that the increase in the ridge thickness contributes 97% of the wavelength shift, whereas the change in the ridge width contributes only 3%, thus indicating a high sensitivity of the BSW mode due to its evanescent nature. Note that when the deposition of the toluene layer or the ridge size increases, the resonant wavelength of the photonic laser red-shifts linearly, but the amount of the wavelength shifts is just 12 % compared to that of the BSW laser. According to these investigations, we conclude that, although it is difficult to distinguish between the sensing mechanisms including the sorption of toluene vapor and the sorption followed by the swelling effect of the SU-8 polymer, a high sensitivity for the BSW lasers could still be obtained.

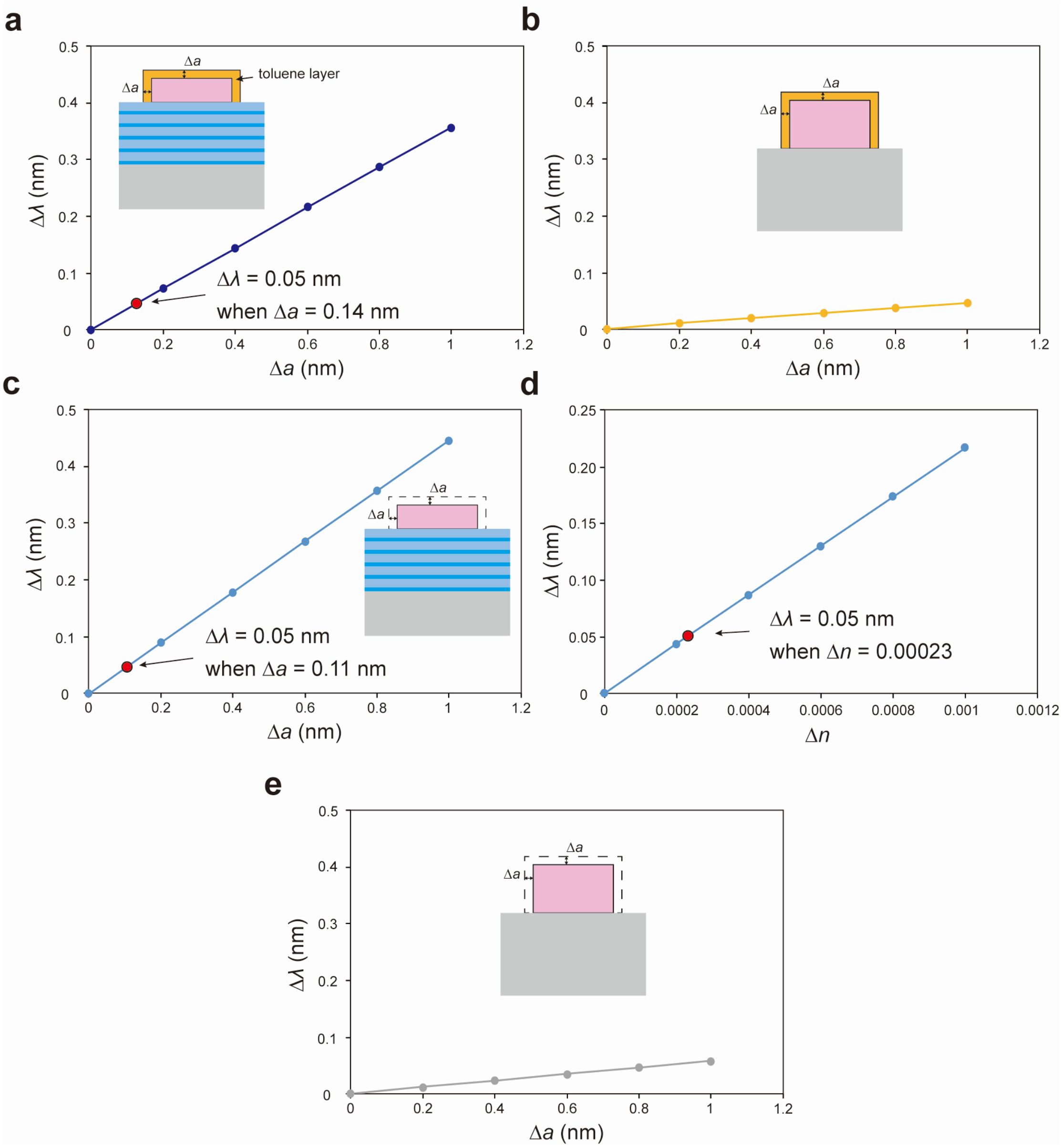

Supplementary Figure 24. The simulated wavelength shifts of (a) the BSW ring lasers and (b) the photonic ring lasers (d = 520 nm) with additionally deposited toluene layer (n = 1.50, considering only the sorption of toluene vapor) onto the surface of the SU-8 polymer ridge. (c, d) The simulated wavelength shifts of the BSW ring lasers with respect to (c) the increase in ridge size Δa and (d) the increase in refractive index Δn (assuming the absorption of toluene vapor without a change in size), simulated separately. (e) The simulated wavelength shifts of the photonic ring lasers (d = 520 nm) with respect to the increase in ridge size Δa. The ring diameter for both BSW laser and photonic laser is 100 µm. The BSW platform comprises five TiO_2/SiO_2 pairs having respective thicknesses of 82 nm/170 nm.

Supplementary References

1. Oulton, R. F. *et al.* Plasmon lasers at deep subwavelength scale. *Nature* **461**, 629-632 (2009).
2. Lu, Y. J. *et al.* Plasmonic nanolaser using epitaxially grown silver film. *Science* **337**, 450-453 (2012).
3. Zhang, Q. *et al.* A room temperature low-threshold ultraviolet plasmonic nanolaser. *Nat. Commun.* **5**, 4953 (2014).
4. Sidiropoulos, T. P. H. *et al.* Ultrafast plasmonic nanowire lasers near the surface plasmon frequency. *Nat. Phys.* 10, 870-876 (2014).
5. Yu, H. C. *et al.* Organic-inorganic perovskite plasmonic nanowire lasers with a low threshold and a good thermal stability. *Nanoscale* **8**, 19536-19540 (2016).
6. Chou, Y. H. *et al.* High-operation-temperature plasmonic nanolasers on single-crystalline aluminum. *Nano Lett.* **16**, 3179-3186 (2016).
7. Wang, S. *et al.* Oulton, R. F., Ma, R. M. Unusual scaling laws for plasmonic nanolasers beyond the diffraction limit. *Nat. Commun.* **8**, 1889 (2017).
8. Zhu, W. *et al.* Surface plasmon polariton laser based on a metallic trench Fabry-Perot resonator. *Sci. Adv.* **3**, e1700909 (2017).
9. Bermúdez-Ureña, E. *et al.* Plasmonic waveguide-integrated nanowire laser. *Nano Lett.* **17**, 747-754 (2017).
10. Wu, Z. Y. *et al.* All-inorganic $CsPbBr_3$ nanowire based plasmonic lasers. *Adv. Opt. Mater.* **6**, 1800674 (2018).
11. Keshmarzi, E. K., Tait, R. N., Berini, P. Single-mode surface plasmon distributed feedback lasers. *Nanoscale* **10**, 5914-5922 (2018).
12. Huang, C. *et al.* Formation of lead halide perovskite based plasmonic nanolasers and nanolaser arrays by tailoring the substrate. *ACS Nano* **12**, 3865-3874 (2018)
13. Ho, Y. L., Clark, J. K., Syazwan, A., Kamal, A., Delaunay, J. J. On-chip monolithically fabricated plasmonic-waveguide nanolaser. *Nano Lett.* **18**, 7769-7776 (2018).
14. Li, H. *et al.* Plasmonic nanolasers enhanced by hybrid graphene-insulator-metal structures. *Nano Lett.* **19**, 5017-5024 (2019).
15. Cho, S., Yang, Y., Soljacic, M., Yun, S. H. Submicrometer perovskite plasmonic lasers at room temperature. *Sci. Adv.* **7**, eabf3362 (2021).
16. Xing, D. *et al.* Delaunay, J. J. Metallic nanowire coupled $CsPbBr_3$ quantum dots plasmonic nanolaser. *Adv. Funct. Mater.* **31**, 2102375 (2021).

17. Wang, J. *et al.* The electron-hole plasma contributes to both plasmonic and photonic lasing from $CH_3NH_3PbBr_3$ nanowires at room temperature. *Laser Photon. Rev.* **15**, 2000512 (2021).

18. Kawabe, Y. *et al.* Whispering-gallery-mode microring laser using a conjugated polymer. *Appl. Phys. Lett.* **72**, 141 (1998).

19. Nilsson, D., Balslev, S., Gregersen, M. M., Kristensen, A. Microfabricated solid-state dye lasers based on a photodefinable polymer. *Appl. Opt.* **44**, 4965 (2005).

20. Grossmann, T. et al. Low-threshold conical microcavity dye lasers. *Appl. Phys. Lett.* **97**, 063304 (2010).

21. Grossmann, T. *et al.* Direct laser writing for active and passive high-Q polymer microdisks on silicon. *Opt. Express* **19**, 12, 11451–11456 (2011).

22. Wienhold, T. *et al.* All-polymer photonic sensing platform based on whispering-gallery mode microgoblet lasers. *Lab Chip* **15**, 3800–3806 (2015).

23. Chandrahalim, H., Fan, X. Reconfigurable Solid-state Dye-doped Polymer Ring Resonator Lasers. *Sci. Rep.* **5**, 18310 (2015).

24. Wan, L. *et al.* On-chip, high-sensitivity temperature sensors based on dye-doped solid-state polymer microring lasers. *Appl. Phys. Lett.* **111**, 061109 (2017).

25. Wan, L. *et al.* Demonstration of versatile whispering-gallery micro-lasers for remote refractive index sensing. *Opt. Express* **26**, 5, 5800–5809 (2018).

26. Deng, C. Z., Ho, Y. L., Lee, Y. C., Wang, Z. Y., Tai, Y. H., Zyskowski, M., Daiguji, H., Delaunay, J. J. Two-pair multilayer Bloch surface wave platform in the near- and mid-infrared regions. *Appl. Phys. Lett.* **115**, 091102 (2019).

27. Badugu, R., Mao, J., Zhang, D., Descrovi, E., Lakowicz, J. R. Fluorophore Coupling to Internal Modes of Bragg Gratings. *J. Phys. Chem. C* **124**, 22743−22752 (2020)

28. Descrovi, E. *et al.* Leakage radiation interference microscopy. *Opt. Lett.* **38**, 17, 3374–3376 (2013).

29. Menotti, M., Liscidini, M. Optical resonators based on Bloch surface waves. *J. Opt. Soc. Am. B* **32**, 431–438 (2015).

30. Cegielski, P. J. *et al.* Monolithically integrated perovskite semiconductor lasers on silicon photonic chips by scalable top-down fabrication. *Nano Lett.* **18**, 6915–6923 (2018).

31. Yang, K. Y. *et al.* Bridging ultrahigh-Q devices and photonic circuits. *Nat. Photon.* **12**, 297–302 (2018).

32. Balslev, S., Rasmussen, T., Shi, P., Kristensen, A. Single mode solid state distributed feedback dye laser fabricated by gray scale electron beam lithography on a dye doped SU-8 resist. *J.*

Micromech. Microeng. **15**, 2456–2460 (2005).

33. Zondervan, R., Kulzer, F., Kol'chenko, M. A., Orrit, M. Photobleaching of Rhodamine 6G in poly(vinyl alcohol) at the ensemble and single-molecule levels. *J. Phys. Chem. A* **108**, 1657–1665 (2004).

34. Singh, S., Kanetkar, V. R., Sridhar, G., Muthuswamy, Raja, K. Solid-state polymeric dye lasers. *J. Lumin.* **101**, 285–291 (2003).

35. Rianjanu, A. *et al.* Swelling behavior in solvent vapor sensing based on quartz crystal microbalance (QCM) coated polyacrylonitrile (PAN) nanofiber. *IOP Conf. Ser.: Mater. Sci. Eng.* **367**, 012020 (2018).

36. Saunders, J. E. *et al.* Quantitative diffusion and swelling kinetic measurements using large-angle interferometric refractometry. *Soft Matter* **11**, 8746–8757 (2015).
