# Supplementary material for: High-Q lasing via all-dielectric Bloch-surface-wave platform
Source: Nat Commun. 2023 Oct 13;14:6458. doi: 10.1038/s41467-023-41471-8 (PMC10576087; doi:10.1038/s41467-023-41471-8)
Supplement: Supplementary file 3 — Reporting summary [file 41467_2023_41471_MOESM3_ESM.pdf]

nature research

Corresponding author(s): Jean-Jacques DELAUNAY

Lasing Reporting Summary

Nature Research wishes to improve the reproducibility of the work that we publish. This form is intended for publication with all accepted papers reporting claims of lasing and provides structure for consistency and transparency in reporting. Some list items might not apply to an individual manuscript, but all fields must be completed for clarity.

For further information on Nature Research policies, including our data availability policy, see Authors & Referees.

Experimental design

Please check: are the following details reported in the manuscript?

1. Threshold

Plots of device output power versus pump power over a wide range of values indicating a clear threshold

- [x] Yes — See Figure 3b.
- [ ] No — *Explain why this information is not reported/not relevant.*

2. Linewidth narrowing

Plots of spectral power density for the emission at pump powers below, around, and above the lasing threshold, indicating a clear linewidth narrowing at threshold

- [x] Yes — See Figures 3a.
- [ ] No — *Explain why this information is not reported/not relevant.*

Resolution of the spectrometer used to make spectral measurements

- [x] Yes — See Figure 3c, Figure S8, and Methods Section.
- [ ] No — *Explain why this information is not reported/not relevant.*

3. Coherent emission

Measurements of the coherence and/or polarization of the emission

- [ ] Yes — *State where this information can be found in the text.*
- [x] No — The BSW laser presented in this report is an on-chip optical component designed to be used as a light source in optical integrated circuits and significantly differs from laser devices designed to emit in the free space. The measurements of the emission coherence and polarization would require the fabrication of additional on-chip components connected to the proposed light source to realize an optical integrated circuit which is out of the scope of this study. It is also noted that the reported very narrow spectral width (0.019 nm) guarantees the coherence property of our device (estimated to be larger than 1 cm from the emission spectral width).

4. Beam spatial profile

Image and/or measurement of the spatial shape and profile of the emission, showing a well-defined beam above threshold

- [ ] Yes — *State where this information can be found in the text.*
- [x] No — For the same reason as in the previous section, it is noted that the proposed BSW laser is not a free-space emitting device and so the beam profile of the BSW laser is entirely defined by the BSW lasing structure (the mode is bound on the surface of the chip). The measurement of the spatial shape and profile of the emission on this onchip device is not relevant.

5. Operating conditions

Description of the laser and pumping conditions
Continuous-wave, pulsed, temperature of operation

- [x] Yes — See Methods/Laser performance measurements.
- [ ] No — *Explain why this information is not reported/not relevant.*

Threshold values provided as density values (e.g. W cm^{-2} or J cm^{-2}) taking into account the area of the device

- [x] Yes — See Figures 3a and 3b.
- [ ] No — *Explain why this information is not reported/not relevant.*

6. Alternative explanations

Reasoning as to why alternative explanations have been ruled out as responsible for the emission characteristics
e.g. amplified spontaneous, directional scattering; modification of fluorescence spectrum by the cavity

- [x] Yes — In Figures 3a and 3b, the emission characteristics possess a clear multimode emission (series of ultra-sharp peaks) together with a nonlinear variation of the emission intensity with the pump power. These characteristics cannot be obtained from amplified spontaneous emission, directional scattering, and modification of fluorescence spectrum in a cavity.
- [ ] No — *Explain why this information is not reported/not relevant.*

7. Theoretical analysis

Theoretical analysis that ensures that the experimental values measured are realistic and reasonable<br>*e.g. laser threshold, linewidth, cavity gain-loss, efficiency*	☒ Yes	See Figures 1, 5b, S9, S14, S15, S16, and S17
	☐ No	*Explain why this information is not reported/not relevant.*

8. Statistics

Number of devices fabricated and tested	☒ Yes	See Figures 4a, 5a, and S10.
	☐ No	*Explain why this information is not reported/not relevant.*
Statistical analysis of the device performance and lifetime (time to failure)	☒ Yes	See Figure S22
	☐ No	*Explain why this information is not reported/not relevant.*
